# Beyond the Silicon Plateau: A Convergence of Novel Materials for Transistor Evolution

**DOI:** 10.1007/s40820-025-01898-8

**Published:** 2025-09-15

**Authors:** Jung Hun Lee, Jae Young Kim, Hyeon-Ji Lee, Sung-Jin Choi, Yoon Jung Lee, Ho Won Jang

**Affiliations:** 1 Department of Materials Science and Engineering, Northwestern University, Evanston, IL 60208 USA; 2 Department of Electrical and Computer Engineering, Texas A&M University, College Station, TX 77845 USA; 3 Department of Material Science and Engineering, Research Institute of Advanced Materials, Seoul National University, Seoul, 08826 Republic of Korea; 4 Department of Chemistry, Northwestern University, Evanston, IL 60208 USA; 5 School of Electrical Engineering, Kookmin University, Seoul, 02707 South Korea; 6 Advanced Institute of Convergence Technology, Seoul National University, Suwon, 16229 Republic of Korea

**Keywords:** Modern transistors, Transistor scaling, Alternative semiconductors, 3D integration, Device reliability

## Abstract

As silicon-based transistors face fundamental scaling limits, the search for breakthrough alternatives has led to innovations in 3D architectures, heterogeneous integration, and sub-3 nm semiconductor body thicknesses. However, the true effectiveness of these advancements lies in the seamless integration of alternative semiconductors tailored for next-generation transistors. In this review, we highlight key advances that enhance both scalability and switching performance by leveraging emerging semiconductor materials. Among the most promising candidates are 2D van der Waals semiconductors, Mott insulators, and amorphous oxide semiconductors, which offer not only unique electrical properties but also low-power operation and high carrier mobility. Additionally, we explore the synergistic interactions between these novel semiconductors and advanced gate dielectrics, including high-*K* materials, ferroelectrics, and atomically thin hexagonal boron nitride layers. Beyond introducing these novel material configurations, we address critical challenges such as leakage current and long-term device reliability, which become increasingly crucial as transistors scale down to atomic dimensions. Through concrete examples showcasing the potential of these materials in transistors, we provide key insights into overcoming fundamental obstacles—such as device reliability, scaling down limitations, and extended applications in artificial intelligence—ultimately paving the way for the development of future transistor technologies.

## Highlights

- This review introduces promising semiconductor materials for future transistors, including two-dimensional van der Waals materials, Mott insulators, halide perovskites, and amorphous oxides, with advantages such as clean interfaces, ultra-thin channels, and defect tolerance.
- These materials, when combined with advanced gate dielectrics and next-generation interconnects, offer synergistic solutions to scaling challenges such as carrier scattering, oxide thickness limitations, and interface degradation.
- The review also discusses reliability concerns including thermal instability and leakage current, and explores future applications in artificial intelligence hardware, in-memory computing, and three-dimensional integration.

## Graphical abstract

**Figure:**
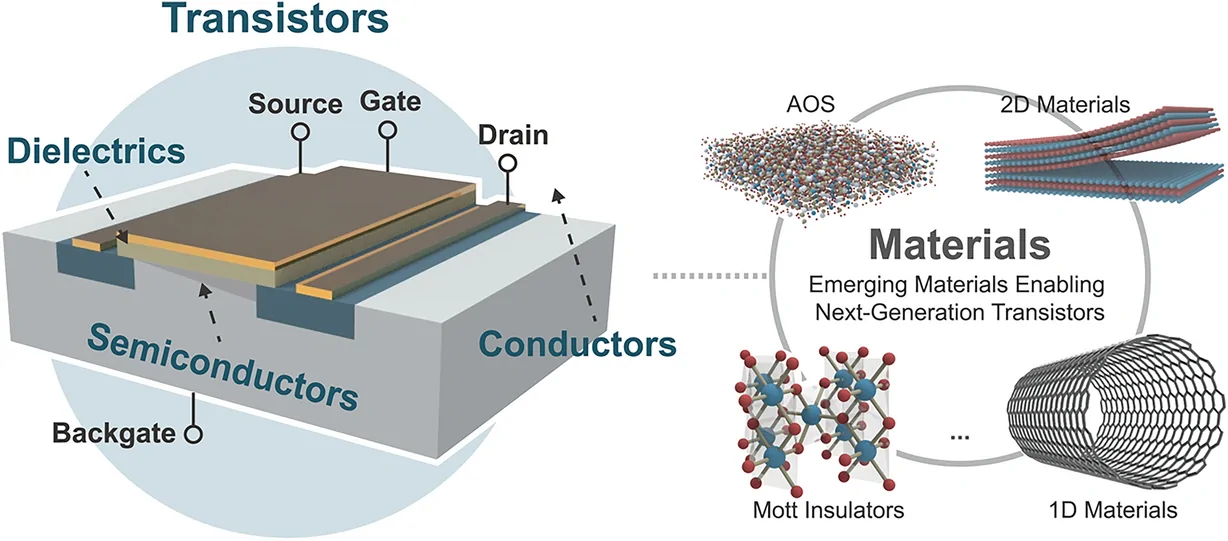

## Introduction

The continuous evolution of transistor technology has driven significant advancements in the semiconductor industry, revolutionizing the field of ranging from the complementary metal–oxide–semiconductor (CMOS) devices to very-large-scale-integration (VLSI) applications [1]. Despite the rapid innovation enabled by advanced transistor technology, the extreme scaling of transistors below the 10 nm gate length and the contacted poly pitch (CPP), which includes both channel length and contact length, has necessitated unprecedented efforts, including achieving ultra-low leakage current [2], sub-1 nm equivalent oxide thickness (EOT) of gate dielectrics [3], and sub-3 nm semiconductor body thickness [4, 5]. Throughout CMOS technology history, research has focused on reducing the body thickness of layers in a transistor [6] alongside shallow dopant implantation in the source–drain regions to prevent active dopant diffusion toward the short channel [7]. However, these approaches have not fundamentally resolved the critical issue posed by short-channel effects (SCEs) in transistors structured with sub-10 nm gate length, resulting in additional challenges, including high contact resistance between semiconductor and source–drain electrodes [8].

To effectively mitigate SCEs in modern transistor technology, several structural advancements have been introduced, including fin field-effect transistors (FinFETs) [9], high-dielectric-constant (*K*) metal gate (HKMG) [10], and silicon-on-insulator (SOI) technology [11]. These innovations have enabled scaling of switching power, reducing operating voltage (*V*_dd_) to approximately 0.8 V and achieving subthreshold swing (S.S.) values approaching the thermionic limit (60 mV dec⁻^1^) [12]. Despite these advancements, significant challenges in transistor scalability have led to the adoption of high-end integration strategies such as 3D monolithic integration [13] or heterogeneous integration [14]. While these integration strategies enhance performance efficiency within a constrained device footprint, they need huge efforts due to the complex circuit designs and processing cost. Rather, these strategies do not fully resolve the intrinsic issue in channel carrier scattering at the semiconductor/gate dielectric interface.

**Fig. 1:**
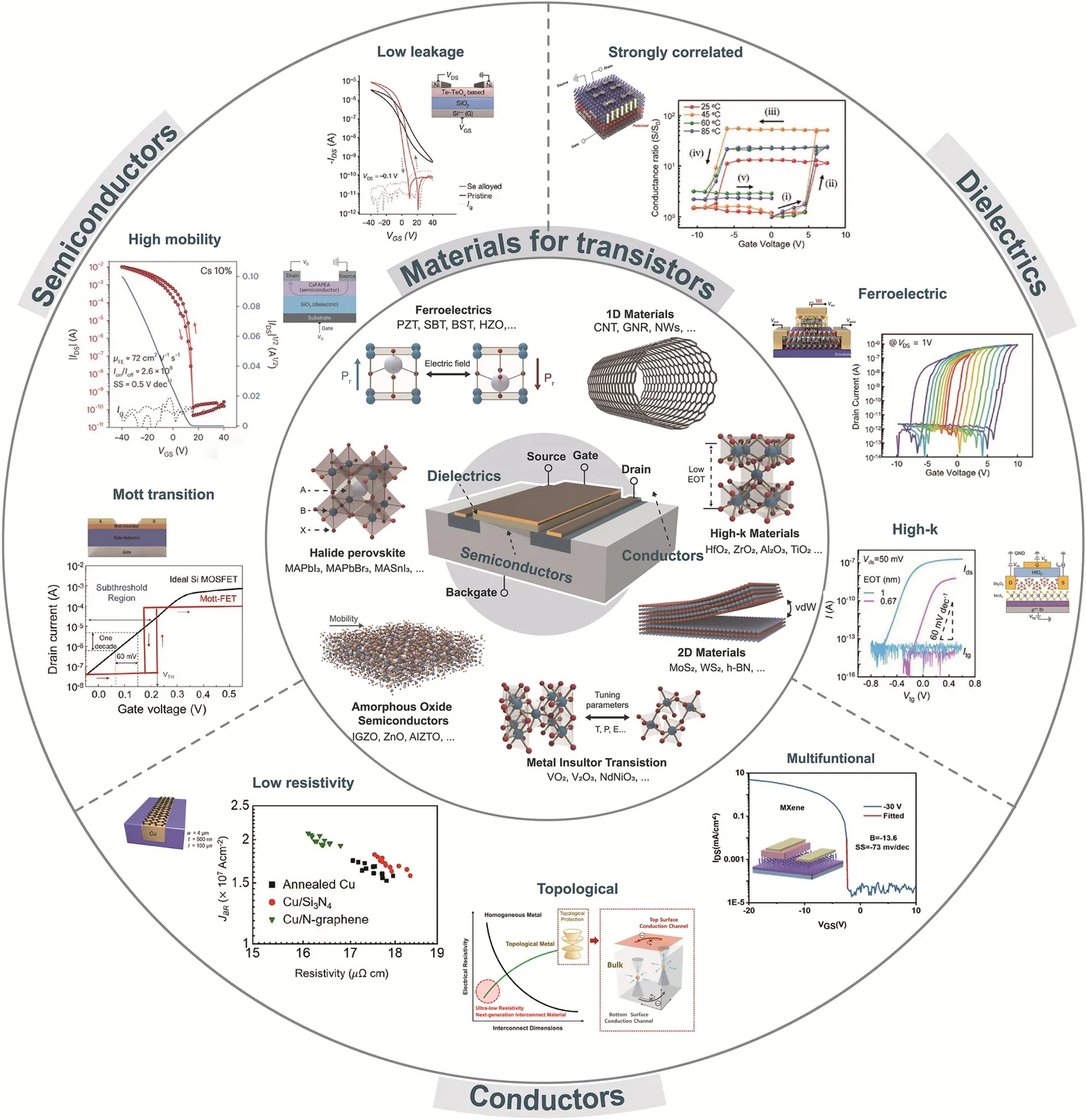
Material properties employed in transistors and their corresponding applications. **a** The materials are allocated to specific components of the transistor, namely **b** semiconductors, **c** dielectrics, and **d** conductors. Transistors constructed on the basis of these material properties exhibit superior performance and multi-functionality. Reproduced with permission [15], Copyright 2024, Wiley–VCH GmbH. Reproduced with permission [16]. CC BY 4.0. Reproduced with permission [17]. CC BY 4.0. Reproduced with permission [18]. Copyright 2022, Wiley–VCH GmbH. Reproduced with permission [19]. Copyright 2022, Wiley–VCH GmbH. Reproduced with permission [20]. Copyright 2023, Springer Nature. Reproduced with permission [21]. Copyright 2022, The Author(s). Reproduced with permission [22]. Copyright 2024, The Author(s). Reproduced with permission [23]. Copyright 2021, Nature Publishing Group

As transistor technology continues to progress, it is crucial to develop fundamental strategies that synergize with high-end integration technologies and structural tuning. One such strategy is to focus on a microscopic channel perspective to promote scattering-free carrier transport. By prioritizing this approach, we can propose a fundamentally innovative but simple solution to drive further advancements in the semiconductor industry. To enhance the channel carrier motion in semiconductors, we need to study alternative semiconductors that can be replaceable for silicon. Figure 1a shows several new classes of semiconductors along with representative high-*K* dielectrics and ferroelectric materials.

In this review, we suggest several alternative semiconductors that can be promising candidates for future scalable transistors. New classes of semiconductors including two-dimensional (2D) van der Waals (vdW) semiconductors, Mott insulators, halide perovskites, and amorphous oxide semiconductors will be introduced with recently reported publications (Fig. 1b). All these materials have shown their unique characteristics including atomically clean surface, ultra-thin body thickness, femto-scale fast switching by gating-induced electron correlation, high carrier mobility, and intrinsic defect tolerance. These properties have been huge advantages for low-powered and highly scalable transistors. Along with the introduced semiconductors, we will address the synergies of new semiconductors/gate insulators including 2D hexagonal boron nitrides, high-*K* dielectrics, and ferroelectric layers (Fig. 1c). By effectively alleviating the EOT degradation issue originating from the increased interfacial defect density (${N}_{\mathrm{it}}$), unfavored nucleation behaviors during the metal oxide growth, degraded ferroelectricity, we can confirm the collaborative usage of dielectrics with new classes of semiconductors. Besides, we will address new metals that can replace interconnects in the back-end-of-line (BEOL). By mitigating the electron scattering on the extremely scaled BEOL region thanks to their unconventional conduction mechanism, several topological semimetals will be introduced (Fig. 1d).

Beyond examining performance innovations in individual transistors, additional concerns are addressed with solutions in Perspectives part. In this part, we care about the device stability issues including thermal instability, leakage current, and long-term degradation—critical concerns in aggressively scaled devices. Additionally, we suggest outlooks for practical application of new materials in modern transistor technologies, in which 3D integration, low-powered devices, and further commercialization toward AI hardware have been necessitated. This review aims to provide material insights relevant to modern transistor technologies, alongside deep understanding the key roles of future materials in a transistor configuration and proposing importance of combined tuning among materials, structures, and systems.

## Evolution of Transistor Technology

**Fig. 2:**
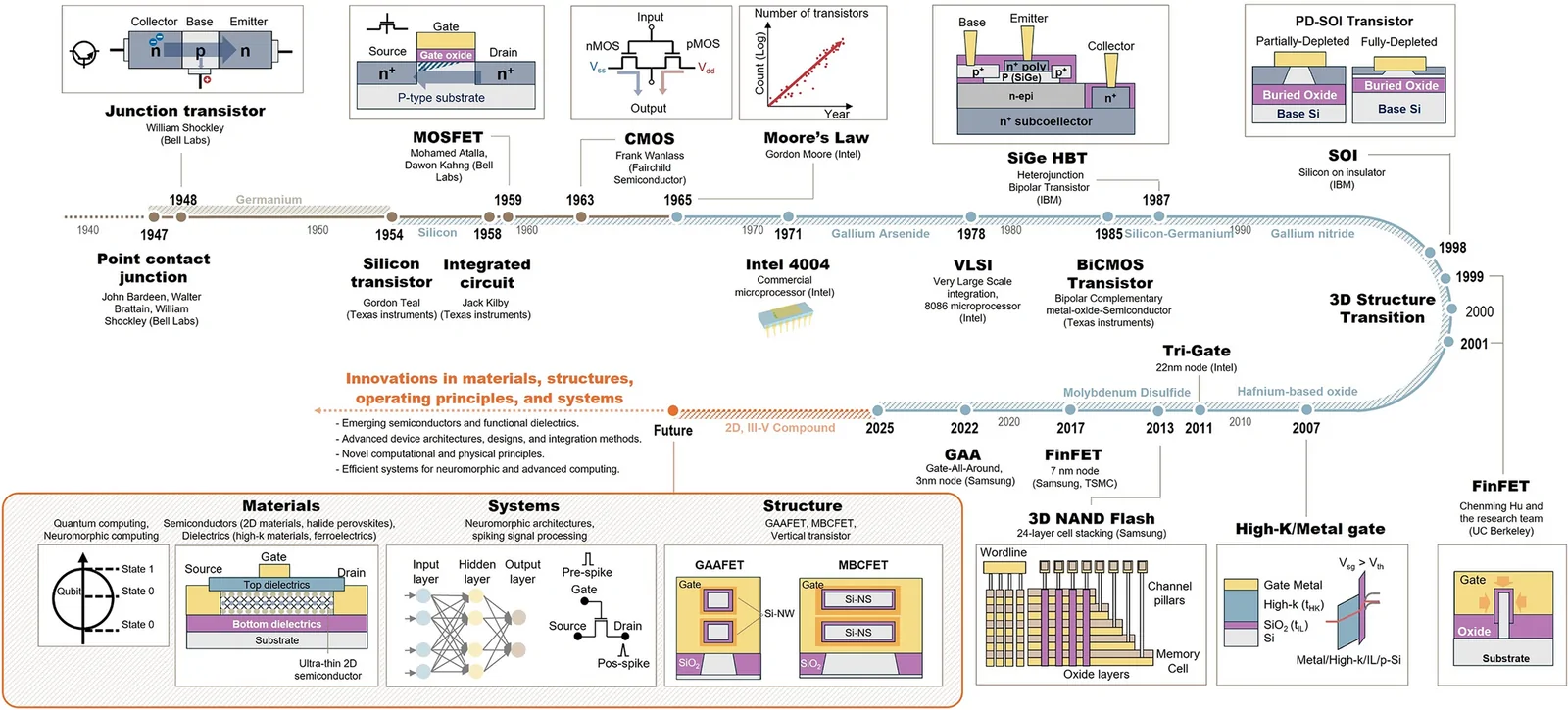
Trends and future perspectives in transistor structures and materials. The progression of transistor technology, initiated in 1947, followed a scaling trend consistent with Moore’s law. While this trend shifted to 3D architectures in the 2000s, scaling challenges prompted the introduction of innovations in materials, operating principles, structures, and systems to further reduce node dimensions beyond traditional scaling limits

The advancement of transistor technology has been the foundation of the semiconductor industry, driving continuous innovation and exponential growth over the past decades (Fig. 2). The first breakthrough occurred in 1947 with the development of the point-contact transistor was developed at Bell Labs, followed by the invention of the bipolar junction transistor (BJT) in 1948, establishing the groundwork for modern semiconductor technology [24]. In 1959, the development of the metal–oxide–semiconductor field-effect transistor (MOSFET) enabled significant scalability and integration. Subsequently, in 1963, the introduction of CMOS technology paved the way for large-scale integration (LSI) [25]. These innovations culminated in the release of the world’s first commercial microprocessor, Intel 4004, in 1971, marking a milestone in modern semiconductor technology [26]. In 1965, Gordon Moore, co-founder of Intel, formulated Moore’s law, predicting that the number of transistors in an integrated circuit would double every 18–24 months. This empirical observation was revised in 1975 to a doubling every two years and became the guiding principle for the semiconductor industry. Since 1993, major semiconductor companies have collaboratively developed the International Technology Roadmap for Semiconductors (ITRS), ensuring the continued progress of Moore’s law and industry’s technological advancements [27]. However, as transistor dimensions shrank below 100 nm, SCEs have become increasingly problematic, posing fundamental limits to traditional planar MOSFET scaling. In the sub-10 nm regime, quantum mechanical tunneling effects cause excessive leakage currents, leading to reduced on-state current (*I*_on_), increased off-state current (*I*_off_), and significant power consumption issues. To address these limitations, the semiconductor industry adopted HKMG technology and introduced FinFET architecture in 2011 [28]. FinFETs, with their three-dimensional gate structure, enhanced electrostatic control over the channel, effectively suppressing leakage currents and improving transistor performance. The gate-all-around (GAA) transistor further advanced transistor design by providing even more precise current control, while 3D NAND flash memory introduced high-density storage capabilities. These 3D integrated structures have minimized interconnect delays and improved power efficiency, playing a crucial role in AI and high-performance computing (HPC) applications [29].

While these advancements have successfully extended the limits of transistor scaling, sustaining Moore’s law has become increasingly challenging due to fundamental physical and economic constraints. Maintaining Moore’s law now requires more than just conventional transistor scaling, as two major obstacles in the form of technological limitations and economic constraints threaten its sustainability. From a technological standpoint, extreme miniaturization below 10 nm leads to quantum tunneling effects, where electrons penetrate the gate oxide, increasing leakage currents and degrading device performance. Furthermore, contact resistance at the source and drain terminals rises as the channel thickness approaches atomic scales, reducing *I*_on_ and overall transistor efficiency. Additionally, heat dissipation in advanced 3D stacked architecture has become a major bottleneck, necessitating innovative thermal management solutions. From an economic perspective, the cost-effectiveness of transistor scaling has significantly declined. Since the 65 nm node in 2006, the cost–benefit ratio of reducing transistor size has diminished. The transition to 22 nm and beyond has required extreme ultraviolet lithography (EUV), an advanced patterning technology that has substantially increased fabrication costs. The semiconductor industry has consolidated around a few dominant players, including Intel, TSMC, Samsung, and GlobalFoundries, as the capital-intensive nature of developing cutting-edge nodes has become a barrier to entry. To address these challenges, the semiconductor industry is shifting focus from pure transistor density scaling to system-level performance optimization through novel materials and 3D integration techniques. Heterogeneous semiconductors have gained attention as potential replacements for traditional silicon-based transistors. 2D materials such as MoS_2_, WS_2_, Mott insulators, HPs, and AOSs exhibit promising properties, including high carrier mobility, atomically thin channels, and defect tolerance, making them strong candidates for next-generation transistors. In parallel, the introduction of high-*K* dielectrics and ferroelectric gate insulators, such as hBN, HfO_2_, and ferroelectric layers, has aimed to reduce the EOT while mitigating interface defect density. Moreover, the transition from planar to 3D monolithic integration is revolutionizing semiconductor architecture. Through-silicon vias (TSV) and compute-in-memory (CIM) technologies are being explored to enhance energy efficiency and reduce data transfer latency, which is particularly crucial for AI-driven workloads and next-generation computing paradigms. The semiconductor industry is continuously exploring new materials, device architectures, and integration methods to overcome the physical limits of Moore’s law. As the industry moves beyond conventional scaling-based transistor improvements, the future of semiconductors will be shaped by heterogeneous integration, 3D stacking, and energy-efficient computing systems.

## Challenges in FET Scaling Technology

### Importance of Low-Power Switching

For nearly two decades since the 1980s, the semiconductor industry predominantly adopted a standard *V*_dd_ of 5 V for digital circuit designs. This was primarily due to the widespread use of bipolar transistor-based digital integrated circuits (ICs) [30]. Even after CMOS technology became the standard architecture for ICs, logic threshold levels remained unchanged to ensure compatibility with existing bipolar logic circuits. However, significant advancements in transistor geometry since the 2000s, particularly as channel lengths shrank below 90 nm [31], enabled the reduction of *V*_dd_ to sub-1 V levels. This voltage scaling has led to substantial reductions in power consumption during CMOS operation. Power consumption (*P*_sw_) scales with the square of the supply voltage (*V*_dd_) as followed Eq. 1, so reducing *V*_dd_ by a factor of 5 lowers *P*_sw_ by 25 times—driving advances in transistor scaling.

1 $$P_{{{\mathrm{sw}}}} = \alpha C_{{\mathrm{L}}} V_{{{\mathrm{dd}}}}^{2} f$$

where α represents the activation coefficient during switching, ${C}_{\mathrm{L}}$ is the load capacitance, and f is the clock frequency. This dramatic improvement in power efficiency has driven the acceleration of transistor scaling technology. While device miniaturization efforts since the 2000s have focused on reducing *P*_sw_, further improvements in switching efficiency require continued voltage scaling. The 2024 IRDS roadmap, for example, projects that *V*_dd_ will be reduced to below 0.7 V by 2028 [32]. Achieving this target necessitates the development of transistors with low S.S., approaching the fundamental thermal limit of 60 mV dec⁻^1^, and a small threshold voltage (*V*th).

To better understand these critical parameters, we need to examine the physical meaning of S.S. and *V*th, along with strategies for minimizing them—particularly in the context of the most widely used transistor in the semiconductor industry: MOSFET. The relationship governing S.S. during transistor switching is given by: For the future target, the most foundational transistor should ultimately have low

2 $${\mathrm{S}}.{\mathrm{S}}. \, ({\mathrm{mVdec}}^{{ - {1}}} ) = { 6}0{\text{ mV}}\left( {{1} + \frac{{C_{{{\mathrm{dep}}}} }}{{C_{{{\mathrm{ox}}}} }}} \right)$$

where *C*_dep_ and *C*_ox_ represent the depletion layer capacitance and gate dielectric capacitance, respectively. To achieve low S.S., parasitic charges must be minimized and high-quality semiconductor/dielectric interfaces ensured to prevent charge scattering [33]. However, high switching speeds also require aggressive channel scaling, especially narrowing the source–drain gap, beyond just optimizing channel materials.

Another crucial parameter in MOSFET operation is the *V*th, which defines the transition from weak to strong inversion in the channel. In an *n* type MOSFET (nMOSFET), applying a positive gate voltage induces band bending at the semiconductor/dielectric interface, leading to channel inversion. The key factors influencing *V*th are described by:

3 $$V{\mathrm{th}}\left( V \right) = V_{{{\mathrm{FB}}}} + \frac{{\sqrt {4Q_{{{\mathrm{dm}}}} qN_{a} \varphi_{{\mathrm{B}}} } }}{{C_{{{\mathrm{ox}}}} }}$$

where *V*_FB_ is the flat-band voltage, *Q*_dm_ is the charge density in the semiconductor, *N*_a_ is the doping concentration of the silicon substrate, ${\varphi }_{\mathrm{B}}$ is the semiconductor bulk potential, and *C*_ox_ is the gate dielectric capacitance. When increasing gate voltage, severe downward band bending is caused at the semiconductor surface. Researchers have explored several strategies to lower *V*th, including increasing *C*_ox_ with high-*K* gate dielectrics, reducing *N*_*a*_, and tuning the flat-band voltage (*V*_FB_) through alternative gate metals. Among these, the combined use of high-*K* dielectrics and lightly doped substrates has emerged as a widely adopted solution. A notable example is TSMC’s demonstration of a standard nMOSFET with a *V*th of 0.847 V [33] using a lightly doped p-type silicon substrate, illustrating the effectiveness of this approach in supporting low-voltage, energy-efficient transistor operation.

### Trend of Transistor Scaling

For low S.S. and *V*th value, therefore, charge scattering—which interferes with the velocity of charge carriers in channel—must be alleviated. However, it is difficult to control the interfacial defects at gate oxide/semiconductor interface and within semiconductor grain boundaries or imperfections without altering the channel geometry. With the revolution of 3D transistor structure starting from FinFET, GAA FET [34, 35], to nanosheet FET [36, 37], the 3 nm gate node technology has been successfully tried and commercialized with the 3D device structure [38]. Although this trend has accomplished a lot of technological advancement in semiconductor field, several challenges remain the mechanical instability from the etching process to form suspended channel [39], channel strain caused by the vertical stress [40, 41], large parasitic capacitance from the 3D structure [42, 43], and the difficulty in achieving a minimum fin width constrained to 4 nm [44]. To move beyond the 3-nm gate-scaled FET, we must conceive next-generation silicon materials that have enough charge mobility at the sub-1 nm thickness. Along with this necessity for new high-mobility semiconducting materials, the high off-current and the resulting low on/off current ratio originally stemmed from the SCE in a highly scalable device should be resolved. For that, we need pertinent hole concentration along with the rich electron concentration, by balancing electron and hole concentration for the low off-current.

**Fig. 3:**
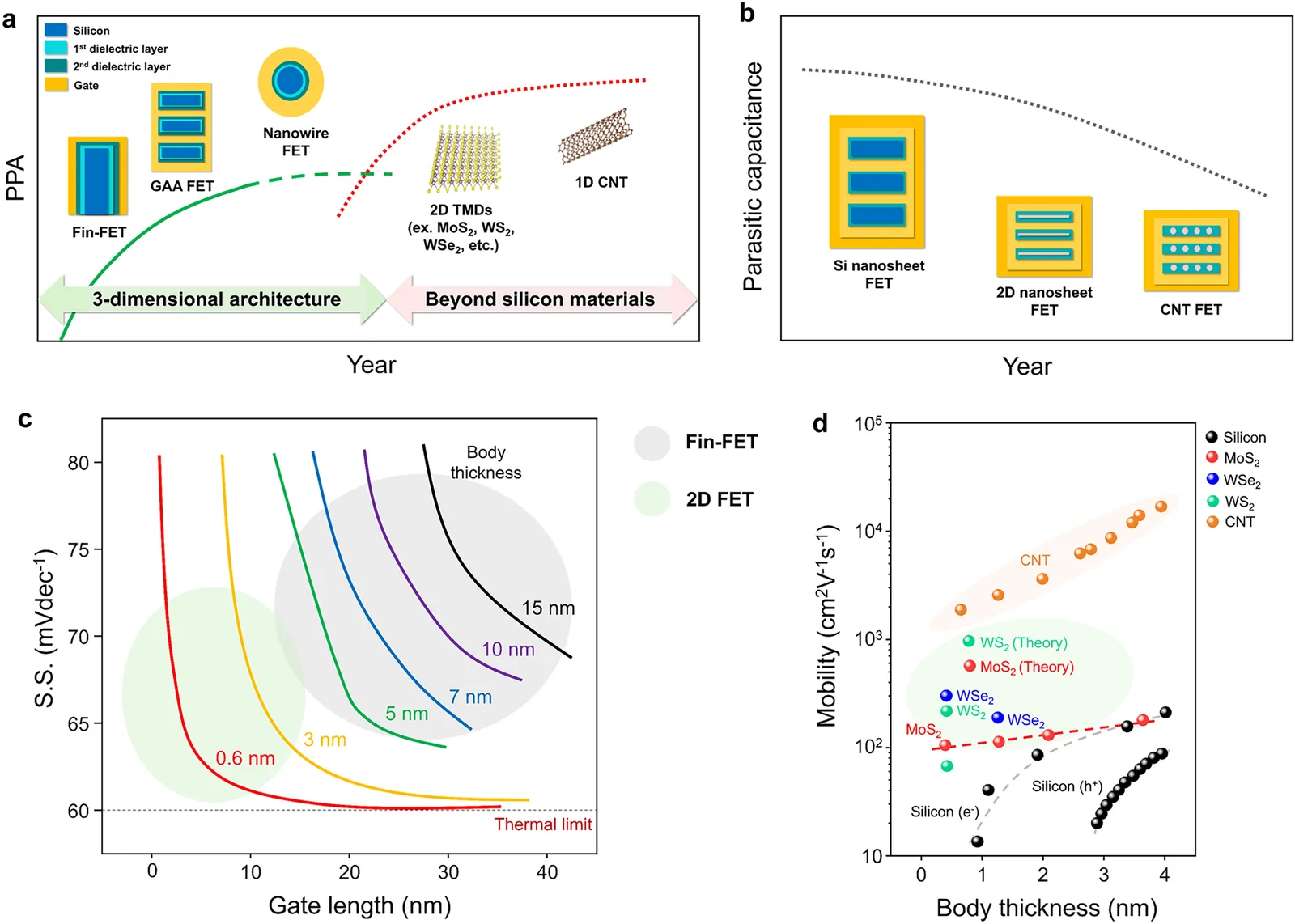
Trends of transistor scaling using beyond-silicon materials in recent semiconductor technologies. **a** Evolution of semiconductor device architecture, progressing from 3D integration to materials beyond silicon. **b** Conceptual schematic representation of advanced transistor scaling techniques, illustrating the decreasing parasitic capacitance as the years passed. **c** S.S. trends depending on the gate length in two representative FET types: FinFET and 2D FET [51]. **d** Exploration of alternative materials for future transistors, such as CNT and 2D TMDs, highlighting their theoretical and experimental performance [52–58]

Even though the power–performance–area (PPA), where it has a trade-off relationship among them, could be improved by the reduced gate node width, much more PPA can be further enhanced using silicon alternatives such as 2D transition metal dichalcogenides (TMDs) or 1D carbon nanotube (CNTs) (Fig. 3a). With these replacements, several logic-level issues have been handled. For example, parasitic capacitance can be alleviated by the application of 2D and 1D materials (Fig. 3b). Given that parasitic capacitance is a key obstacle that impedes the speed and energy efficiency of the aggressively scaled silicon transistors due to higher contact area and reduced oxide body thickness, accounting for over 70% of the total capacitance [45], these low-dimensional semiconductors are advantageous to addressing the power issues. Especially in scaled transistors, trapping sites at dense interfaces—one of the main causes of parasitic capacitance—can be alleviated by the van der Waals gap at 2D material surfaces and by the ballistic transport along the surface of CNTs. Therefore, low-dimensional materials are promising candidates for minimizing parasitic capacitance.

According to the 2023 IRDS roadmap [32], 3D VLSI configured with vertical CFET is targeted for implementation by 2034. As this 3D stacking trend continues, the demand for utilizing 2D materials as channel materials in specialty transistor adds-on is expected to increase significantly. In addition, to prevent performance degradation of the lower FET during device processing, the processing temperature of the upper FET must be limited to below 400 °C. By employing thermally robust 2D materials as the channel material for the lower FET, the thermal budget for the upper FET process can be effectively extended. Along with the material suitability for vertical CFET, it is possible to approach the fundamental limit of S.S., which is 60 mV dec⁻^1^ under room temperature, when applying 2D materials (Fig. 3c). It is because the charge mobility is dramatically enhanced compared to the typical silicon. Specifically, the mobility of monolayer TMD 2D and CNT is approximately 10 and 100 times higher than that of silicon, respectively, when compared at the same thickness scale of around 1 nm [48, 50] (Fig. 3d).

Along with this enhanced mobility, the atomically flat and defect-free surfaces of 2D van der Waals semiconductors—free of dangling bonds—facilitate the formation of clean and trap-free interfaces with compatible 2D dielectric materials such as hBN or layered oxides [46, 47]. While such vdW interfaces offer significant advantages in suppressing interface scattering and enabling super low depletion capacitance (*C*_dep_), it should also be noted that additional interface engineering is essential when integrating conventional 3D high-*K* dielectrics like Al_2_O_3_ or HfO_2_ due to their intrinsic dangling bonds [48]. Through the use of these highly conductive yet defect-free materials with sub-nanometer thicknesses, substantial progress has been made in realizing low-power transistor switching and aggressive electrostatic scaling.

Although there are a lot of advantages in scaling when using 2D and 1D materials in the semiconductor channel of a transistor, it has still many obstacles to apply those in thin-film transistors (TFTs) display application in terms of difficulties in large-scale uniform coating [49], and alignment issue with metal electrodes [50]. Furthermore, considering the values of intrinsic delay time compared between logic and thin TFTs for display (Table 1), more prolonged intrinsic delay time in TFT applications than that in logic applications alludes that relatively strong gate capacitance and contact resistance between semiconductor and metal should be more carefully controlled in the case of low-dimensional semiconductor devices.

**Table 1:** Summary of recent studies about intrinsic time delay of low-dimensional TFTs and logics

Device type	CNT TFT	WS_2_ TFT	WSe_2_ TFT	MoS_2_ TFT	CNT logic	WS_2_ logic	WSe_2_ logic	MoS_2_ logic
Intrinsic time delay	281 ps	30.7 ps	66 ps	0.276 ps	16 ps	0.118 ps	8.96 ns	6.8 ns
References	[59]	[60]	[61]	[62]	[63]	[64]	[65]	[66]

### Scaling Limitation and Strategies Based on New Materials

**Fig. 4:**
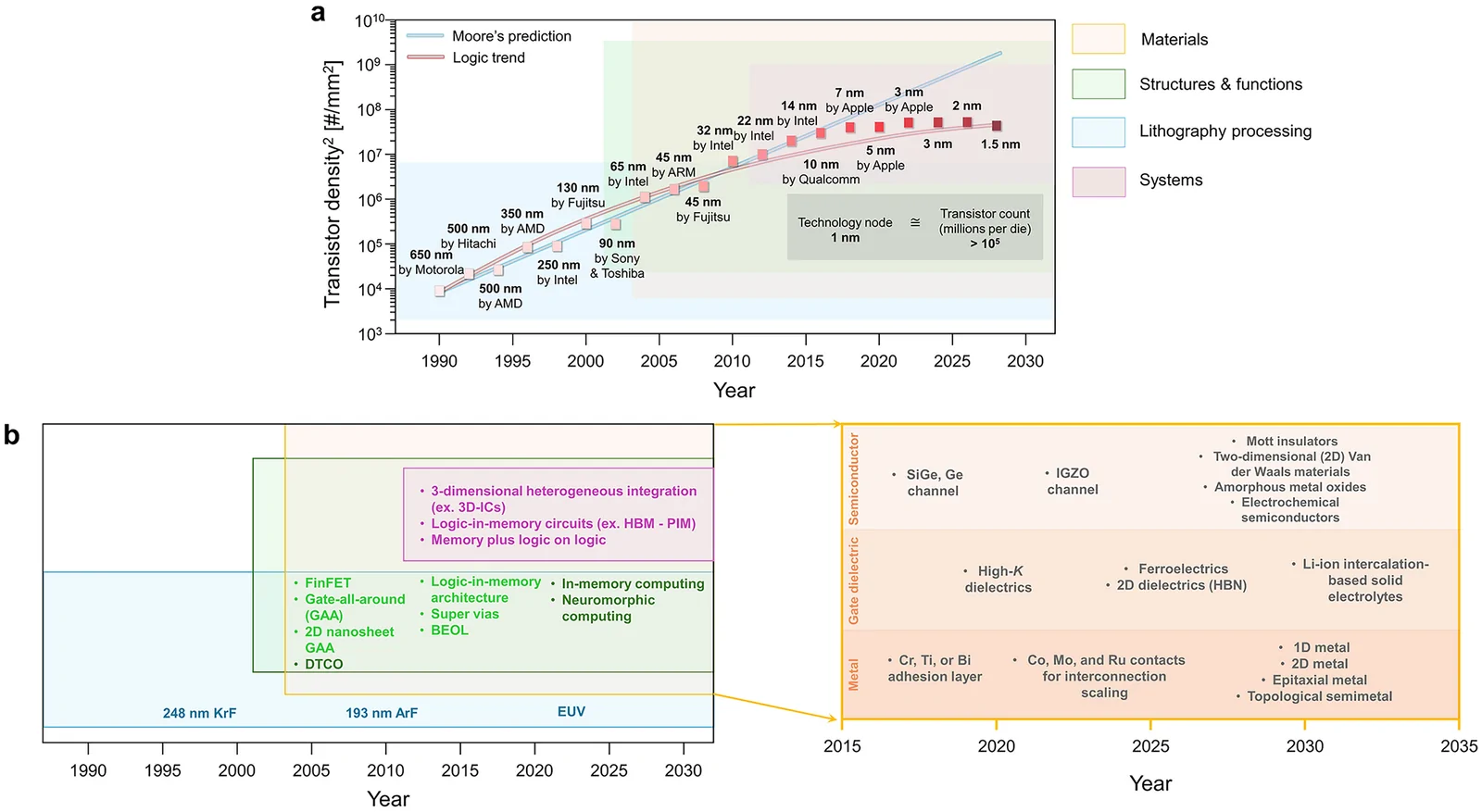
Scaling limitations of recent FETs. **a** Transistor density trends over the past 35 years and projections for the next 5 years, highlighting the discrepancy between Moore’s prediction and the actual logic scaling trend [104, 105]. Each channel length and the fabrication company is denoted at each trend. A scaling criterion dividing actual transistor device size to virtual transistor size according to Moore’s predictions has emerged around 2010. **b** Evolution of materials [15, 18, 77, 79, 84] for recent transistors device structures [36, 72, 106, 107] and functions [33, 108, 109], lithography processes [110], and systems [72, 111, 112] over the past 40 years. Yellow, green, blue, and red regions represent trends in semiconductor materials, structures and functions, lithography, and system advancements, respectively. Among the several factors to influence scalability, detailed materials trend for metal contacts, gate dielectrics, and semiconductor layers are summarized from 2015 to 2035

Although Moore’s law has long served as a guiding principle for transistor scaling, a growing gap between Moore’s predictions and actual trends in logic device scaling has highlighted its limitations in recent decades (Fig. 4a). Since 2010, a critical criterion has emerged, distinguishing between actual transistor sizes and virtual scaling predictions. This shift spurred a paradigm change, addressing transistor scaling through advancements in materials, device functions, fabrication processes, and system evolution. These four key domains have recently led to distinct trajectories within the semiconductor technology landscape (Fig. 4b). Developing diverse solutions beyond nanopatterning technologies has become increasingly important for the future of transistors, particularly due to the limitation of EUV lithography. Despite notable progress, including successful production of 3 nm chips using EUV lithography by Samsung, TSMC, and Intel, several challenges have emerged in terms of the inefficiency of multi-patterning processes [67, 68] and environmental impact of EUV [69]. Consequently, relying solely on next-generation patterning technologies beyond EUV light sources is not the most effective strategy for continuing device scaling. Instead, researchers are exploring versatile approaches that leverage advancements across the four domains.

In addition to research and development (R&D) revolutions of nanopatterning technologies, new system algorithms and computing functionalities are increasingly integrated into transistor-based hardware. Examples include 3D heterogeneous integrated architecture [70, 71], logic-in-memory circuits [72, 73], and memory plus logic on logic device systems [74, 75]. These innovations have enabled the proposal and validation of new computing paradigms, demonstrating compatibility with other systems and enhancing overall performances. Additionally, a new methodology for time-to-market process—technology co-optimization (DTCO) [76]—has emerged by various semiconductor industries. By introducing one of methods to increase PPA, meaningful circulation between processing and marketing has been enabled. While system-level developments are promising, critical trends in FET technology between 2015 and 2035 have been highlighted by the evolution of critical components such as metal contacts, gate dielectrics, and semiconductor layers. This trend underscores the increasingly complex demands of next-generation devices, which extend beyond simple tuning using only semiconducting material.

For the semiconductor layers, transistor devices have been applied by SiGe or Ge [77, 78], indium–gallium–zinc oxide (IGZO) [79, 80], Mott insulators [15, 18], 2D vdW materials [81, 82], HPs [83, 84], and various amorphous metal oxides [17, 85]. For the gate dielectric layers, key materials include high-*K* dielectrics [86], ferroelectrics [80], 2D dielectrics like hBN [87, 88], and lithium (Li)-ion intercalated solid electrolytes [89, 90]. Meanwhile, molybdenum (Mo) [91], bismuth (Bi) [92, 93], and antimony (Sb) [94–96] have been adopted as an adhesion layer beneath Au or Pt source/drain electrodes to achieve ohmic contact at metal/semiconductor junctions. Additionally, emerging materials such as one-dimensional (1D) metals [97, 98], 2D metal [99–101], or Weyl semimetals (WSMs) [102, 103] are being investigated as an alternative gate material to reduce resistivity.

Ultimately, it is critical to understand how to apply these materials effectively to different transistor configurations. In the following sections, we will comprehensively categorize and discuss the suitability of each material for three key components: semiconductors, gate dielectrics, and metal contacts. We will also analyze their impact on transistor performance and their specific functionalities.

## Next-Generation Semiconductors

### 2D vdW Semiconductors

The synthesis of 2D vdW semiconductors plays a pivotal role in optimizing their structural, electronic, and interfacial properties for high-performance transistor applications. Various synthesis strategies, categorized into bottom-up and top-down approaches, offer distinct advantages depending on the specific requirements of material quality, scalability, and device integration [113].

Bottom-up synthesis methods enable precise control over the atomic structure and crystallinity of 2D materials, making them particularly suitable for high-performance electronic and optoelectronic applications. Chemical vapor deposition (CVD) [113–115] and metal–organic chemical vapor deposition (MOCVD) [116–118] are widely employed techniques for the synthesis of high-quality monolayer and few-layer 2D films. These methods rely on the precisely controlled reaction of vapor-phase precursors under a defined temperature and pressure regime, enabling layer-by-layer growth with exceptional crystallinity and uniformity. The resulting 2D nanosheets exhibit excellent electronic properties, including high carrier mobility and minimal defect density, making them ideal candidates for next-generation transistors. While these methods have enabled the synthesis of wafer-scale 2D films with lateral dimensions extending up to 2–4 inches [119–121], several challenges persist in achieving industrial scalability. Maintaining uniform thickness and crystallinity across large areas remains a critical issue due to variations in precursor flux, thermal gradients, and substrate-induced strain, which can lead to structural inhomogeneities and defect formation at grain boundaries. Addressing these limitations is imperative for the successful integration of 2D materials into scalable electronic and optoelectronic technologies.

Epitaxial growth techniques further refine the bottom-up synthesis of 2D semiconductors by enabling the deposition of atomically precise layers with well-defined crystallographic orientations. Molecular beam epitaxy (MBE) [122–125] utilizes ultra-high-vacuum conditions to direct atomic or molecular beams onto a substrate, allowing for the formation of monolayers or few-layer structures with extreme purity and controlled thickness. This method is particularly advantageous for fundamental studies and high-performance electronic applications where defects must be minimized. van der Waals epitaxy (vdWE) extends the capabilities of epitaxial growth by leveraging weak van der Waals interactions to enable the synthesis of 2D materials on substrates with lattice mismatches. Unlike conventional epitaxy, which relies on strict lattice registry, vdWE permits the growth of heterostructures with minimal interfacial strain, thereby expanding the range of compatible substrate choices. Nevertheless, similar to CVD and MBE, epitaxial growth techniques face limitations in large-area scalability and are primarily employed for specialized applications where material quality takes precedence over throughput.

For applications requiring high-quality bulk 2D materials, chemical vapor transport (CVT) [126, 127] and flux growth [128] methods provide an effective means of synthesizing pristine crystals that can be subsequently exfoliated into thin layers. In CVT, volatile transport agents, such as halogens, facilitate the vapor-phase migration of precursor species, enabling the controlled crystallization of high-purity bulk materials. This method is widely utilized for the synthesis of layered 2D semiconductors with well-defined stoichiometry and structural integrity. Flux growth, on the other hand, involves dissolving precursor materials in a high-temperature solvent, whereupon controlled cooling induces crystal precipitation. This technique enables the synthesis of large, high-quality single crystals with minimal structural defects, making them particularly valuable for fundamental studies and device fabrication requiring bulk material integrity. Although CVT and flux growth yield high-purity bulk crystals, their direct applicability to wafer-scale integration remains limited. Mechanical exfoliation (ME), while effective for obtaining high-quality monolayers and few-layer flakes, is inherently restricted in scalability. In contrast, liquid-phase exfoliation (LPE) [129–132] enables the production of solution-processable 2D inks, facilitating the fabrication of large-area thin films through deposition techniques such as spin coating, printing, and solution shearing. This makes LPE-derived films particularly advantageous for scalable transistor applications and flexible electronics. Consequently, CVT- and flux-grown bulk crystals serve as essential precursors for solution-processing techniques, bridging the gap between high-quality material synthesis and large-area device fabrication.

The synthesis of 2D van der Waals semiconductors via bottom-up approaches provides a versatile pathway for tailoring material properties to enhance transistor performance. While methods such as CVD, MOCVD, and epitaxy enable precise structural control, their scalability remains a challenge for industrial-scale integration. Bulk crystal growth techniques, including CVT and flux growth, offer an alternative route to high-quality layered materials but require additional processing for thin-film applications. Representative studies highlighting wafer-scale growth techniques and fabrication methods of 2D materials [133], including chemical vapor deposition [134, 135], metal–organic CVD (MOCVD) [136], atomic layer deposition (ALD) [137], epitaxial/hypotaxial growth [138, 139], and printing methods approaches [140, 141], serve as valuable resources toward both foundational principles and recent advancements relevant to the scalable integration of 2D semiconductors. The continued development of scalable, high-purity synthesis strategies remains essential for the widespread adoption of 2D materials in next-generation nanoelectronic devices.

**Fig. 5:**
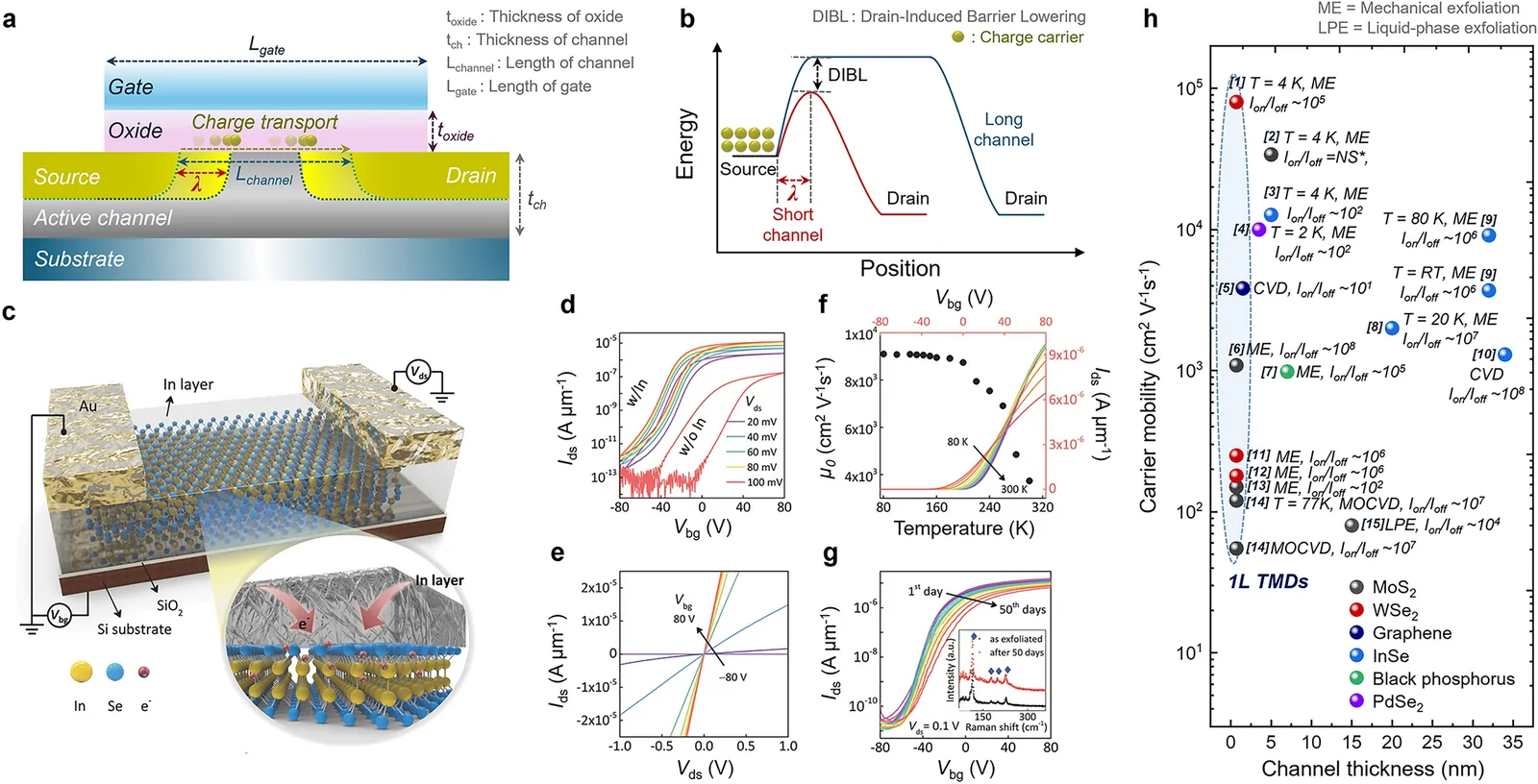
2D vdW semiconductors. **a** Schematic representation of FETs at the extreme scale, depicting critical structural parameters for charge carrier transport. **b** Energy band diagram illustrating the long-channel and short-channel configurations of a transistor, highlighting DIBL and characteristic screening length (λ). **c** Schematic of a back-gate InSe FET device, encapsulated with an indium (In) layer to protect and dope the surface. The inset provides details of the SCTD process [144]. **d** Transfer characteristics of a layered InSe FET (32 nm In encapsulation) measured under varying drain–source voltages (*V*_ds_), compared with a device without In encapsulation at *V*_ds_ = 0.1 V [144]. **e** Output characteristics of the InSe FET (32 nm In encapsulation) at different back-gate voltages (*V*_bg_) with 20 V increments [144]. **f** Temperature-dependent transfer characteristics of the InSe FET (32 nm In encapsulation), demonstrating carrier mobility trends as a function of temperature. **g** Stability assessment of the InSe FET (32 nm In encapsulation) over time, showcasing the evolution of transfer characteristics, Raman spectra, carrier mobility, and hysteresis loop window size over 50 days [144]. **h** Carrier mobility as a function of channel thickness for two-dimensional transistors based on ME [136, 144, 151–158], liquid-phase exfoliation [141], CVD [159], and MOCVD [160] methods. NS* not specified. Reproduced with permission from [144]. Copyright 2018, Wiley

2D vdW semiconductors have emerged as transformative materials for next-generation transistor technologies, offering unique advantages over conventional bulk semiconductors in terms of electrostatic control, dimensional scaling, and charge transport efficiency. The layered structure of vdW materials enables the realization of an atomically thin film at the monolayer limit (*t*_channel_ < 1 nm) without the formation of surface dangling bonds, thus minimizing surface roughness scattering and suppressing defect-induced trap states. These intrinsic properties allow 2D materials to effectively mitigate short-channel effects (SCEs), which pose a significant challenge in the aggressive downscaling of conventional FETs. In traditional semiconductor FETs (Fig. 5a), as the channel length (*L*_channel_) decreases to the nanometer scale, the electrostatic control exerted by the gate becomes increasingly compromised due to the intensified electrostatic coupling between the source and drain electrodes. This results in pronounced SCEs, including drain-induced barrier lowering (DIBL), *V*th roll-off, and an increase in S.S.. These effects collectively degrade the transistor’s switching behavior, leading to excessive off-state leakage currents, reduced *I*_on_/*I*_off_ ratios, and unpredictable electrical characteristics, ultimately undermining device reliability and energy efficiency. In contrast, 2D vdW semiconductors inherently mitigate these SCEs due to their atomically thin channel geometry, which significantly enhances electrostatic gate control. The ultra-thin body of 2D materials ensures that the entire channel remains within the gate’s electrostatic influence, effectively suppressing charge-sharing effects from the drain and minimizing the depletion region encroachment that typically exacerbates DIBL (Fig. 5b). This strong electrostatic confinement preserves a well-defined potential barrier between the source and drain, allowing for precise carrier modulation even at deeply scaled channel lengths. Furthermore, unlike conventional semiconductors that suffer from surface states and dangling bonds at the semiconductor–dielectric interface, 2D vdW materials exhibit pristine surfaces with van der Waals bonded layers, eliminating trap-induced Coulombic scattering. This absence of interface trap states reduces carrier scattering at the semiconductor–dielectric interface, facilitating ballistic or quasi-ballistic charge transport. As the gate length (*L*_g_) is reduced below 5 nm, the simultaneous reduction in channel length and body thickness imposes severe constraints on the electrostatic integrity and carrier transport of Si-based transistors. Although bulk silicon possesses an intrinsic electron mobility of approximately 1000 cm^2^/Vs, this value deteriorates drastically in ultra-scaled geometries due to pronounced short-channel effects (SCEs) such as drain-induced barrier lowering (DIBL) and direct source-to-drain tunneling, ultimately making it increasingly difficult for silicon to sustain reliable switching performance at such dimensions. In contrast, atomically thin 2D materials, when implemented in lateral device architectures [142, 143], enable aggressive channel length scaling down to the sub-1 nm regime while maintaining strong gate control and suppressing SCEs. Wu et al*.* [143], demonstrate sub-1 nm channel length transistors (“side-wall transistors”) based on 2D lateral heterostructures, exhibiting a subthreshold swing as low as 117 mV dec^−1^, and an *I*_on_*/I*_off_ ratio of ~ 10^5^, all on a 2-inch wafer platform. These findings underscore the unique potential of 2D semiconductors in extending Moore’s law beyond the physical limits of silicon. Consequently, 2D FETs demonstrate superior electrostatic integrity, low off-state leakage, and enhanced energy efficiency, making them promising candidates for further sub-10 nm transistor technologies.

Among the diverse class of 2D van der Waals semiconductors, indium selenide (InSe) has emerged as a promising 2D semiconductor for high-performance FETs due to intrinsically high carrier mobility, tunable bandgap, and strong gate modulation capabilities. Despite its advantageous electrical properties, InSe suffers from air instability, which leads to oxidation and defect formation, and contact resistance ultimately degrading device performance. To overcome these limitations, surface charge transfer doping (SCTD) approach using an indium (In) encapsulation layer was developed, significantly enhancing both the electrical performance and long-term operational stability in InSe FETs [144]. As illustrated in Fig. 5c, mechanically exfoliated InSe nanosheets (10–13 layers, ~ 9.21 nm thickness) were transferred onto Si/SiO_2_ (300 nm) substrates in a back-gate FET configuration. The In-encapsulated (w/In) InSe FET exhibits a dramatic enhancement in drain current (*I*_ds_) and gate tunability, eliminating the severe current suppression observed in unencapsulated (w/o In) devices (Fig. 5d). This confirms SCTD-driven n-type doping, which reduces the Schottky barrier at the source/drain contacts and facilitates seamless carrier injection. Further evidence of contact resistance reduction is seen in Fig. 5e, where the output characteristics (*I*_ds_–*V*_ds_) reveal a near-ohmic behavior at *V*_bg_ = ± 80 V, ensuring efficient charge transport owing to a lowered Schottky barrier at metal–semiconductor interfaces. Figure 5f exhibits temperature-dependent field-effect mobility (*µ*_0_), which peaks at an impressive ~ 9100 cm^2^ V⁻^1^ s⁻^1^ at 80 K. This increase in mobility at low temperatures is largely due to the suppression of phonon scattering, which dominates at higher temperatures. For 2D semiconductors, long-term stability is just as critical as high performance. The hysteresis-free transfer curves in Fig. 5g demonstrate that In encapsulation prevents charge trapping, ensuring consistent *V*th and *I*_ds_ over 50 days of operation. Raman spectroscopy (Inset, Fig. 5g) confirms minimal structural degradation, underscoring the effectiveness of In encapsulation in suppressing environmental-induced defects.

While most 2D vdW semiconductors inherently exhibit n-type behavior, certain materials, such as black phosphorus (BP) and tungsten diselenides (WSe_2_), have been identified as promising p-type or ambipolar candidates. Achieving stable and efficient p-type conduction in 2D transistors is essential for realizing complementary logic circuits, optoelectronic devices, and neuromorphic computing architectures. The following studies illustrate how band structure engineering, doping, and solution-processing strategies are transforming 2D FETs beyond traditional *n* type transport. BP [145, 146] has emerged as a promising candidate for next-generation nanoelectronics due to its intrinsic *p* type transport, high hole mobility, and layer-dependent bandgap tunability, distinguishing it from conventional *n* type-dominated 2D TMDs. Unlike TMDs, which require chemical doping or electrostatic gating to achieve *p* type conduction, BP naturally supports hole transport, making it an essential material for all-2D complementary logic circuits. Furthermore, its tunable bandgap, which ranges from 2.0 eV in monolayer BP (black phosphorene) to ~ 0.3 eV in bulk, enables precise control over charge carrier dynamics, facilitating its integration into high-performance transistors, near-infrared optoelectronics, and logic circuits [147]. The electronic transport properties of BP exhibit a non-monotonic dependence on thickness, dictated by the interplay of bandgap evolution, electrostatic gate efficiency, and charge carrier scattering mechanisms. In the monolayer limit, BP retains a large direct bandgap (~ 2.0 eV) due to quantum confinement effects, which enhances p-type conduction by stabilizing the valence band edge while suppressing thermally excited electron transport. However, several limitations arise in this ultra-thin regime: 1) Strong quantum confinement and surface effects. The quantum confinement-induced bandgap widening increases the hole effective mass, limiting carrier mobility. Additionally, the strong influence of surface interactions makes monolayer BP highly sensitive to environmental conditions and dielectric effects, posing challenges for device stability and performance. 2) Coulombic scattering and interface roughness effects. As monolayer BP lacks sufficient charge screening, it is highly susceptible to charged impurity scattering, particularly from the underlying dielectric. This leads to mobility degradation despite strong electrostatic gate modulation. While monolayer BP provides excellent gate tunability, its mobility remains constrained by strong carrier–impurity interactions and surface scattering. Notably, the monolayer BP is more air-sensitive at the monolayer level than thicker layers. As BP thickness increases, its bandgap gradually narrows to ~ 0.6–0.7 eV at ~ 10 nm, significantly enhancing charge carrier mobility. This thickness range offers optimal transport characteristics, as demonstrated by BP FETs fabricated using ~ 10-nm-thick mechanically exfoliated BP [148]. Few-layer BP FETs exhibit one of the highest hole mobilities (~ 1000 cm^2^ V⁻^1^ s⁻^1^) at room temperature among p-type 2D semiconductors, significantly surpassing most TMD-based transistors. The additional layers provide better charge screening, reducing Coulombic impurity scattering and improving transport efficiency. The reduced bandgap in few-layer BP results in stronger band dispersion, lowering the hole effective mass and facilitating efficient charge transport. At ~ 10 nm thickness, BP maintains strong gate control while minimizing interfacial disorder, achieving an optimal balance between mobility, bandgap tunability, and electrostatic gating efficiency.

In another study, they demonstrated the fabrication of wafer-scale arrays of molybdenum disulfide (MoS_2_)-based transistors through a commercial slot-die printing process. Utilizing tailored inks of MoS_2_ nanosheets and sodium-embedded alumina (SEA), we achieve precise deposition of the semiconductor and gate dielectric layers, respectively. The resulting transistors achieve remarkable charge carrier mobilities of 80.0 cm^2^ V⁻^1^ s⁻^1^ in FET measurements and 132.9 cm^2^ V⁻^1^ s⁻^1^ in Hall measurements at room temperature, with uniformity across the wafer. The high mobility observed in the MoS_2_-on-SEA devices is primarily attributed to the SEA dielectric layer, which promotes band-like charge carrier transport through the well-percolated MoS_2_ thin-film networks. The SEA layer’s high dielectric constant enables efficient electron mobility by fostering van der Waals sheet-to-sheet contacts with energetically flat electronic properties, enhancing uniformity across the five-inch wafers. Notably, the high-*K* dielectric properties of SEA are advantageous for solution-processed MoS_2_ thin films, comparable to those of hafnium oxide (HfO_2_) or aluminum oxide (Al_2_O_3_), which are typically used to enhance field-effect mobility. Additionally, they successfully integrate these transistors into logic circuits such as NOT, NAND, NOR, and SRAM, demonstrating a viable pathway for large-area, solution-processed 2D electronics. This approach provides a promising route to high-performance, flexible electronics based on 2D materials at commercial scales [141].

To enhance the electrical performance and stability of 2D materials, chemical doping and functionalization are widely utilized, as their effects are particularly impactful in 2D systems. In MoS_2_ transistors, for instance, TFSI (trifluoromethanesulfonimide) chemical treatment has demonstrated substantial improvements, with electron mobility increasing from 4.7 to 18.9 cm^2^ V⁻^1^ s⁻^1^ and a fivefold enhancement in subthreshold swing. Previous studies suggest that TFSI molecules passivate sulfur vacancies on the basal plane of MoS_2_ nanosheets, which function as carrier trapping sites, thereby reducing the density of charge traps. Furthermore, TFSI acts as a Lewis acid, effectively lowering the charge density of MoS_2_ by withdrawing electrons. This reduction in charge density significantly lowers the off-current, thus maximizing the *I*_on_–*I*_off_ ratio and enhancing device performance [132, 141]. Building on this, T. Zou et al*.* present an effective doping strategy using bromine (Br_2_) to enhance the hole mobility and operational stability in p-type transistors based on 2D WSe_2_. While TFSI treatment has shown significant performance gains for n-type MoS_2_ by passivating sulfur vacancies and lowering charge density, Br_2_ doping offers a similarly transformative effect for p-type WSe_2_ transistors. Specifically, Br_2_ doping increases the field-effect hole mobility from ~ 0.004 cm^2^ V⁻^1^ s⁻^1^ (undoped) to 27 cm^2^ V⁻^1^ s⁻^1^ and achieves an *I*_on_/*I*_off_ current ratio exceeding 10^7^, with excellent stability under repeated cycling, bias stress, and switching conditions. Density functional theory (DFT) calculations reveal that Br_2_ molecules adsorb onto the WSe_2_ surface, covering up to 25% and creating shallow acceptor states near the valence band maximum. The doping reduces contact resistance and enhances charge transport by lowering the Schottky barrier at metal contacts to ~ 80 meV, compared to the 1 eV barrier typically seen in undoped WSe_2_. This allows efficient hole injections, as demonstrated by temperature-dependent measurements, where Br_2_ doping achieves low activation energy (*E*_a_) values (32-85 meV) across the channel. This improved charge injection and transport are attributed to reduced channel resistance and minimized inter-flake hopping. The enhanced charge injection and transport in Br_2_-doped WSe_2_ transistors are primarily due to a substantial reduction in channel resistance and minimized inter-flake hopping. Br_2_ has proven to be the most effective p-type dopant of WSe_2_, significantly increasing hole concentration and lowering channel resistance compared to other doping treatments like ultraviolet (UV)–O_3_, HBr, TFSI, and PEDOT treatments. Br_2_’s small molecular size allows it to penetrate inter-flake boundaries and contact regions, facilitating uniform and comprehensive doping across nanoflake films, which overcomes the limitations of conventional molecular dopants typically restricted to surface adsorption [149].

The two-dimensional van der Waals materials, including InSe, BP, molybdenum telluride (MoTe_2_), tin sulfide (SnS_2_), and others, are highly sensitive to air or moisture, necessitating careful handling under inert environments or the use of passivation layers to prevent degradation and maintain their properties for applications. Among these materials, BP stands out due to its high charge carrier mobility, tunable bandgap, and strong anisotropic properties. However, it rapidly degrades in ambient conditions due to its reactivity with oxygen and water, resulting in a substantial loss of electronic performance. To address this instability, covalent functionalization with aryl diazonium salts has proven to be an effective method for stabilizing BP. This approach forms robust phosphorus–carbon (P–C) bonds that effectively passivate BP, preserving its morphology and preventing degradation for over three weeks in ambient exposure. The chemical functionalizaiton was achieved by immersing few-layer BP (~ 10 nm thick, prepared by ME) in a solution of 4-nitrobenzene diazonium (4-NBD) tetrafluoroborate salts. The functionalized BP exhibits robust morphology and stable atomic force microscopy (AFM) profiles even after extended air exposure, confirming effective passivation. Note that air-sensitive materials, when exposed to ambient conditions, rapidly form an oxide layer, leading to an increase in thickness and surface roughness. This functionalization also improves the electronic properties of BP-based FETs. Specifically, the p-type doping introduced by aryl diazonium chemistry enhances both the *I*_on_*/I*_off_ and hole carrier mobility of BP FETs. Higher doping levels achieved through increased aryl diazonium concentration reduce current modulation as BP becomes degenerately doped, while lower concentrations (~ 1 µM) allow for controlled doping with minimal *I*_off_ increase. The functionalization rate is influenced by the reduction potential of the aryl diazonium substituent, allowing precise control over BP’s electronic characteristics. Consequently, aryl diazonium chemistry provides a promising approach for enhancing both the stability and performance of BP-based devices [150].

Figure 5h illustrates the correlation between carrier mobility and channel thickness in 2D van der Waals semiconductors synthesized via various techniques, including ME, LPE, CVD, and MOCVD. The data show that mechanically exfoliated few-layer graphene and InSe achieve the highest mobility, exceeding 10^4^ cm^2^ V⁻^1^ s⁻^1^ at cryogenic temperatures (*T* = 4 K, 20 K), surpassing theoretical expectations. Ultimately, when precisely engineered, 2D semiconductors have the potential to bridge the gap between high-performance transistor technologies and scalable device manufacturing, facilitating their integration into next-generation electronic and optoelectronic systems.

### Halide Perovskites

**Fig. 6:**
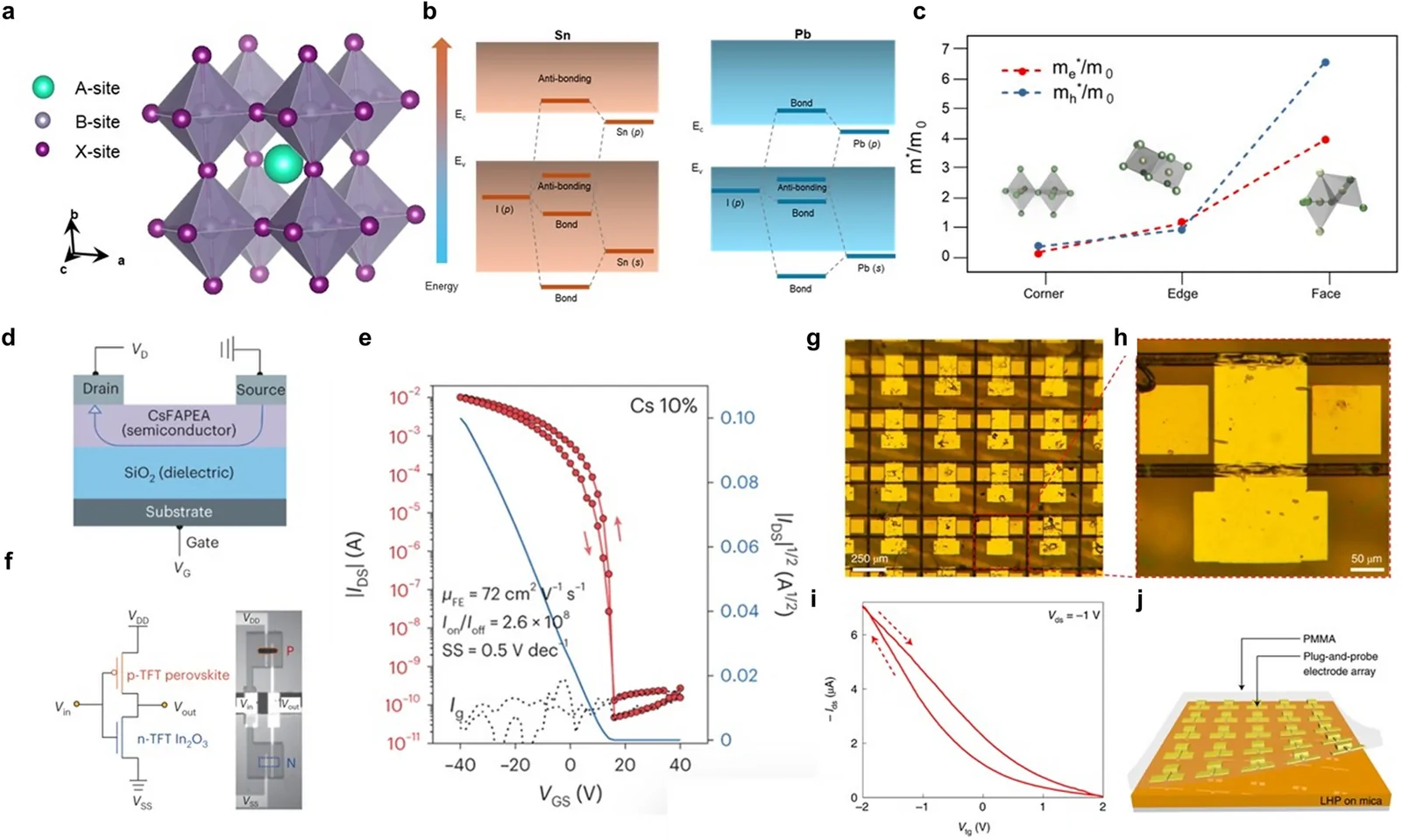
Halide perovskite transistors. **a** Crystal structure of halide perovskites. **b** Band positions of Sn- and Pb-based halide perovskites. **c** Carrier effective mass of halide perovskites as a function of increasing the octahedron connectivity. Reproduced with permission from [181]. Copyright 2022 American Chemical Society. **d** Schematic structure of a Sn-based perovskite transistor. **e** Transfer characteristics of a CsFAPEA-based transistor. **f** Structural scheme and optical image of the inverter. Reproduced with permission from [16]. CC BY 4.0. **g** Optical microscopy image and **h** magnified view of a selected region showing the fabricated top-gated perovskite transistor array. **i** Transfer curve of a perovskite transistor. **j** Schematic illustration of a plug-and-probe electrode array on a mica substrate. Reproduced with permission from [179]. Copyright 2022, Springer nature

Recent advances in HP semiconductors, primarily in optoelectronic applications, have renewed attention in their use for transistors. HPs are emerging as promising semiconductor candidates to replace silicon in advanced transistor technologies. Their unique material characteristics, such as low-temperature processability, defect tolerance, and ambipolar transport, offer fundamental advantages over conventional silicon, which suffers from short-channel effects, high contact resistance, and S.S. limitations in the sub-5 nm regime [161, 162]. Notably, the intrinsic ionic motion in perovskites enables mixed ionic–electronic conduction, allowing for tunable switching behavior and neuromorphic functionalities not achievable in rigid covalent semiconductors like silicon [163]. These attributes, combined with the ability to engineer electronic properties via compositional tuning, position HPs as a compelling platform for next-generation low-power, high-density, and multi-functional transistors [164]. MHPs are a class of compounds with an ABX_3_ crystal structure, where A represents a monovalent organic or inorganic cation, B is a divalent metal cation such as Pb^2+^ or Sn^2+^, and X corresponds to a halide anion, including Cl^–^, Br^–^, or I^–^. As illustrated in Fig. 6a, the [BX_6_]^4–^ octahedra form a corner-sharing network within a cubic or tetragonal lattice, while A-site cations occupy the interstitial spaces, stabilizing the structure [165]. These materials exhibit long charge carrier diffusion lengths, high charge mobility, and defect tolerance, making them attractive candidates for transistor applications. Unlike conventional semiconductors, perovskites can be processed at low temperatures, offering a cost-effective and scalable alternative to traditional silicon-based thin-film transistors. The electronic properties of HPs are highly sensitive to their synthesis conditions, necessitating controlled processing strategies to ensure device stability, scalability, and performance [166]. The ability to process MHPs at low temperatures offers a significant advantage over conventional polycrystalline silicon thin-film transistors, which require thermal processing above 400 °C. Two primary deposition strategies exist for MHP thin films: one-step deposition and two-step deposition [167].

In one-step deposition, all precursor components such as MAI and PbI_2_ are co-deposited onto a substrate via solution-processing or vapor-phase methods. This approach is attractive due to its simplicity and rapid film formation, making it suitable for commercial production. However, rapid crystallization often leads to poor film morphology and high defect densities. To address these issues, anti-solvent engineering is employed, where a non-coordinating solvent is introduced during spin coating to promote uniform nucleation and suppress defect formation [168]. Additionally, dual-source or single-source evaporation techniques enable vapor-phase deposition of perovskite precursors under vacuum, forming high-purity thin films with excellent crystallinity, albeit at the cost of increased fabrication complexity and lower deposition rates [169].

In two-step deposition, precursor materials are sequentially deposited. Typically, a metal halide such as PbI_2_ is first deposited as a thin film, followed by exposure to an organic halide vapor or solution to induce perovskite conversion. This method allows for better control over crystallinity, thickness, and uniformity, albeit at the expense of increased processing steps and longer fabrication times. Furthermore, precise interface engineering is crucial to minimize interlayer defects that could degrade transistor performance. Solution-based processing techniques, such as spin coating, blade coating, and inkjet printing, enable large-area deposition with cost-effective manufacturing. However, residual solvents can lead to performance degradation, requiring post-deposition annealing and additive engineering to optimize film morphology and electronic properties. While vacuum-based techniques offer superior stability, they require further optimization for scalable manufacturing [158].

The charge transport characteristics of MHPs are strongly influenced by their chemical composition, phase stability, and structural connectivity [166]. The B-site cation plays a crucial role in determining the semiconductor type, with Pb-based perovskites typically exhibiting ambipolar transport behavior, whereas Sn-based perovskites strongly favor p-type conduction due to the presence of Sn vacancies (Fig. 6b) [170]. Sn-based perovskites offer higher hole mobility but suffer from excessive carrier concentrations, making field-effect modulation challenging. In contrast, Pb-based perovskites benefit from stronger spin–orbit coupling, resulting in enhanced band dispersion and tunable transport properties [162, 171]. In addition to composition, octahedral connectivity significantly impacts charge mobility. Corner-sharing octahedral networks minimize carrier effective mass, leading to superior charge transport compared to edge- or face-sharing configurations (Fig. 6c) [172]. This structural advantage makes corner-sharing perovskites highly desirable for high-mobility FETs, where isotropic charge transport is required [161].

Despite their promising properties, MHPs face stability challenges related to ion migration. Halide ion diffusion leads to hysteresis in electrical characteristics and *V*_th_ shifts in FETs. Additive engineering offers a practical approach to suppressing defect formation. For instance, SnF_2_ additives mitigate Sn vacancy formation, improving p-type transport stability, while RbCsFAMA cation engineering has been shown to minimize hysteresis in Pb-based perovskites [132, 173]. Additionally, halide substitution including Br^–^ or Cl^–^ for I^–^ promotes enhanced crystallization and passivates iodine vacancies, further reducing defect densities. Interfacial engineering is equally critical in optimizing MHP transistor performance. The semiconductor/dielectric and semiconductor/electrode interfaces strongly influence charge injection, mobility, and hysteresis behavior. Using MoO_x_ hole injection layers has been demonstrated to enhance hole mobility in 2D (PEA)_2_SnI_4_ transistors, while employing high-*K* dielectrics like HfO_2_ has enabled the realization of hysteresis-free perovskite transistors [174]. Addressing interfacial stability remains a key challenge for the practical deployment of MHP-based electronics.

HP-based transistors were first demonstrated in 1999 by IBM using (PEA)_2_SnI_4_ as a p-type TFT channel material [175]. However, material instability hindered further development until 2014, when renewed interest emerged with the demonstration of MAPbI_3_-based transistors and self-doped ambipolar transport characteristics [176, 177]. Recently, high-performance CsSnI_3_-based TFTs have achieved *µ*_h_ exceeding 50 cm^2^ V⁻^1^ s⁻^1^ and *I*_on_/*I*_off_ of 10^8^, surpassing conventional amorphous silicon (a-Si) and oxide semiconductors in charge transport efficiency [166]. Furthermore, Zhu et al*.* demonstrated a high-performance transistor based on A-site engineering (Fig. 6d). The device shows an exceptionally high hole mobility of 70 cm^2^ V⁻^1^ s⁻^1^ and an *I*_on_*/I*_off_ of 10^8^ (Fig. 6e) [16]. The optimization of the Cs molar ratio was key to fabricating high-quality perovskite films, enabling the successful integration of NAND and NOR logic gates (Fig. 6f) [16]. These advancements highlight the potential of MHP transistors not only for logic applications but also for low-power electronics. Beyond their conventional use in FET applications, MHPs have demonstrated significant potential for neuromorphic computing due to their unique mixed ion–electron conduction properties [163]. Ion migration in perovskites mimics synaptic plasticity, where halide ions and metal vacancies act as dynamic charge trapping sites, enabling multi-level resistive states for hardware-based AI learning. Unlike conventional resistive random-access memory (RRAM) devices, 3-terminal neuromorphic transistors based on perovskites allow for decoupled signal processing and weight updating, enhancing learning stability and energy efficiency. Jeong et al*.* demonstrated a CsPbBr_3_ transistor with a ferroelectric gate that successfully emulated synaptic behavior, offering a promising path toward hardware-integrated artificial intelligence systems [178].

Given their tunable band structures and ionic transport characteristics, perovskites also present opportunities for hybrid integration with existing semiconductor technologies. Combining MHPs with 2D materials such as MoS_2_ and WS_2_ enables high-performance, energy-efficient neuromorphic computing architectures, while plug-and-probe integration techniques facilitate CMOS-compatible device fabrication (Fig. 6g, h) [179]. This approach involves the simultaneous vdW integration of high-*K* dielectrics and contacts in a single step. They fabricated top-gated CsPbBr_3_ transistors showing low operating voltage (Fig. 6i) and the transistor array using the vdW plug-and-probe method offering a reliable path toward scalable fabrications (Fig. 6j). Moreover, heterostructures integrating perovskites with TMDs have demonstrated bio-inspired optical adaptation, paving the way for intelligent image sensors and AI-driven vision systems [180].

HPs exhibit unique advantages for next-generation semiconductor applications, bridging the gap between high-performance electronic materials and scalable, low-cost processing techniques. Advances in synthesis control, interfacial engineering, and hybrid integration have significantly improved material stability and device performance, making them viable candidates for low-power computing, neuromorphic devices, and flexible electronics. While long-term stability and CMOS compatibility remain critical challenges, ongoing innovations in material design and device fabrication hold great promise for realizing perovskite-based electronics in future semiconductor technologies.

### Mott Insulators

For ultra-fast switching and low-power operation in transistors, Mott insulators have emerged as promising semiconductor candidates. Despite being termed “insulators,” these materials exhibit unique electronic properties in transistors due to electron correlation effects induced by external stimuli such as gate voltage, light exposure, temperature, or pressure [15]. This correlation-driven phenomenon enables metal–insulator transition (MIT) behavior in Mott insulators, leading to high resistivity contrast and significant variations in charge density. The dramatic modulation of charge density via bandgap formation/collapse has been actively explored in semiconductor channels, giving rise to a new class of devices known as Mott transistors. In contrast to silicon, where scaling below 5 nm is hindered by short-channel effects and fundamental thermal limits on subthreshold swing, Mott systems offer steep-slope switching (sub-10 mV dec⁻^1^) and retain robust electrostatic integrity even at atomic dimensions [182]. Their high intrinsic carrier density, coupled with negligible Thomas–Fermi screening, facilitates gate coupling at nanometric thicknesses without compromising *I*_on_/*I*_off_ ratios or mobility [183]. These features collectively position Mott insulators as compelling candidates for beyond-CMOS logic, where ultra-low-power operation, aggressive dimensional scaling, and neuromorphic functionality converge. Various Mott insulators, including NiO [184], NbO_2_ [185], ternary chalcogenide compounds [182], and VO_2_, exhibit MIT behavior. However, this review will focus specifically on VO_2_-based Mott transistors. As a representative Mott insulator, VO_2_ undergoes an MIT in which the insulating monoclinic (M_1_) phase transforms into the metallic rutile (R) phase when subjected to temperatures above 68 °C [186] or high surface charge densities exceeding 10^15^ cm⁻^2^ under a strong electrostatic field [187].

**Fig. 7:**
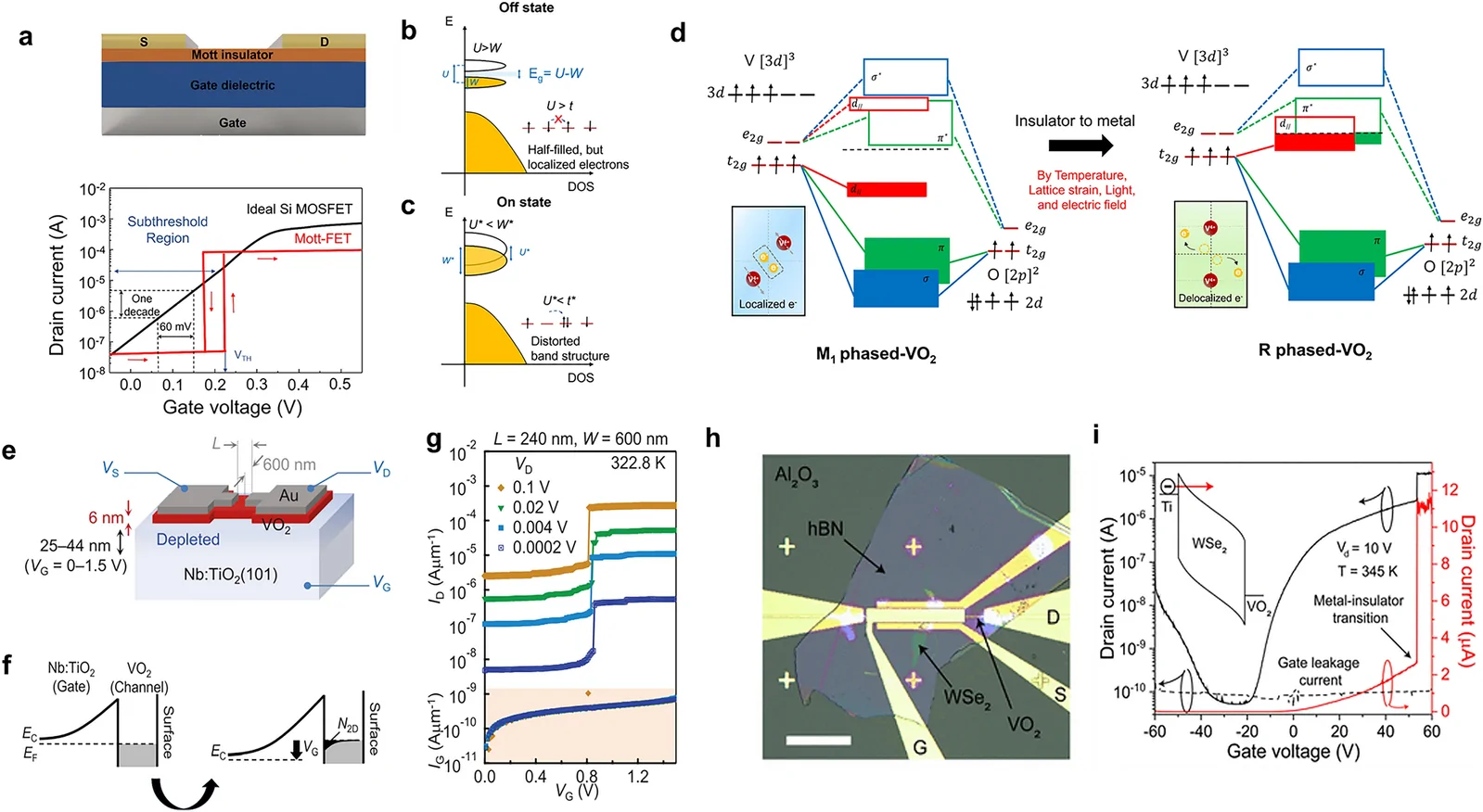
Mott transistor. **a** Schematic illustration of a typical Mott transistor with a bottom-gate top-contact (BGTC) structure, demonstrating abrupt switching behavior with a small S.S. value. The electronic structure of the Mott insulator is depicted in the **b** off state and **c** on state. Reproduced with permission [15], Copyright 2024, Wiley–VCH GmbH. **d** Changes in the band structure of VO_2_ as it transits from the M_1_ insulating phase to the R metallic phase under various perturbations, including temperature, lattice strain, light, and electric field. **e** A Three-terminal VO_2_ transistor using a Nb-TiO_2_ gate electrode. **f** Band diagram of the VO_2_/Nb-TiO_2_ contact during the operation of the device shown in Fig. 7e under gate voltage application. **g** Transfer characteristics at various drain voltages, ranging from 0.0002 to 0.1 V, measured at 322.8 K. Reproduced with permission [190], Copyright 2023, Nature Publishing Group. **h** Multilayer WSe_2_ transistor with a VO_2_ contact electrode as the drain and hBN as gate dielectric. **i** Transfer curve of the device in Fig. 7 h measured at 345 K, along with the gate leakage current (*I*_g_). Reproduced with permission [191], Copyright 2019, American Chemical Society

Beyond the conventional MIT in VO_2_, its ability to achieve ultra-fast switching within just a few picoseconds [188] and operate with extremely low writing energies below 100 fJ [168] makes VO_2_-based transistors highly promising for low-power logic applications. Additionally, VO_2_ offers a pathway to overcoming the scaling limits of conventional semiconductors due to its negligible Thomas–Fermi screening effect [15]. Notably, even at dimensions below 10 nm, electron delocalization in VO_2_ preserves high conductivity comparable to a bulk film [187], making it an excellent candidate for ultra-scaled semiconductor devices. As depicted in Fig. 7a, the typical switching behavior of Mott transistor is introduced. This performance is distinctly different from that of conventional FET. By inducing much lower S.S. value that overcomes the typical thermionic limit (60 mV dec⁻^1^), the abrupt switching becomes possible. Shukla et al*.* reported a super steep-slope transistor in which VO_2_ is connected to the drain electrode of an InGaAs quantum well FinFET [189]. All these examples of fast switching in the case of VO_2_ transistors are attributed to the abrupt electron delocalization in the channel, allowing MIT behavior to be observed during switching. Figure 7b, c provides further insight into this switching mechanism using density of states (DOS) diagram. Unlike conventional FETs, where channel formation occurs through thermal excitation of carriers by an external gate field, Mott FETs undergo band structure modifications that facilitate electron delocalization. Since the electronic state, in other words band structure, transitions along with the structural phase change (Fig. 7d), this process is inherently rapid and allows for a lower *V*th.

Leveraging the unique and intriguing electronic phenomena in VO_2_, various types of transistors have been developed. Yajima et al*.* reported an unconventional transistor configuration in which a depletion region between Nb-doped TiO_2_ and VO_2_ served as a virtual gate dielectric (Fig. 7e) [190]. The VO_2_ layer was epitaxially grown on an Nb-doped TiO_2_ substrate with a precisely controlled thickness of 6 nm. As shown in Fig. 7f, the interface between VO_2_ channel and the substrate formed a depletion region extending several tens of nanometers. This depletion layer acted as an insulating barrier between the gate and the channel. When a positive gate voltage was applied to the substrate, the depletion layer expanded, attracting a high concentration of electrons. This substantial electron accumulation induced a metallic state in VO_2_, enabling low-power isothermal switching at an extremely low *V*_ds_ (0.004 V) while achieving an *I*_on_/*I*_off_ ratio of 10^3^ (Fig. 7g). VO_2_ has also been integrated into 2D FET as drain electrode. Yamamoto et al*.* demonstrated a device utilizing a TMD 2D material, WSe_2_, with an hBN gate dielectric, where a VO_2_ nanowire was employed as the drain electrode (Fig. 7h). Notably, charge carriers in 2D channel were ambipolarly modulated and exhibited abrupt magnification, resulting in an *I*_on_/*I*_off_ current ratio of 10^3^ and S.S. of 60 mV dec⁻^1^ (Fig. 7i). This current amplification was attributed to the MIT behavior in VO_2_, induced by gate-mediated self-heating. As illustrated in these studies, VO_2_, whether utilized as a semiconductor or a drain electrode, facilitates ultra-fast switching enabled by gate-induced MIT behavior. This gate tunability in VO_2_-based transistors highlights their potential as promising candidates for low-power transistor applications.

### Amorphous Oxide Materials

**Fig. 8:**
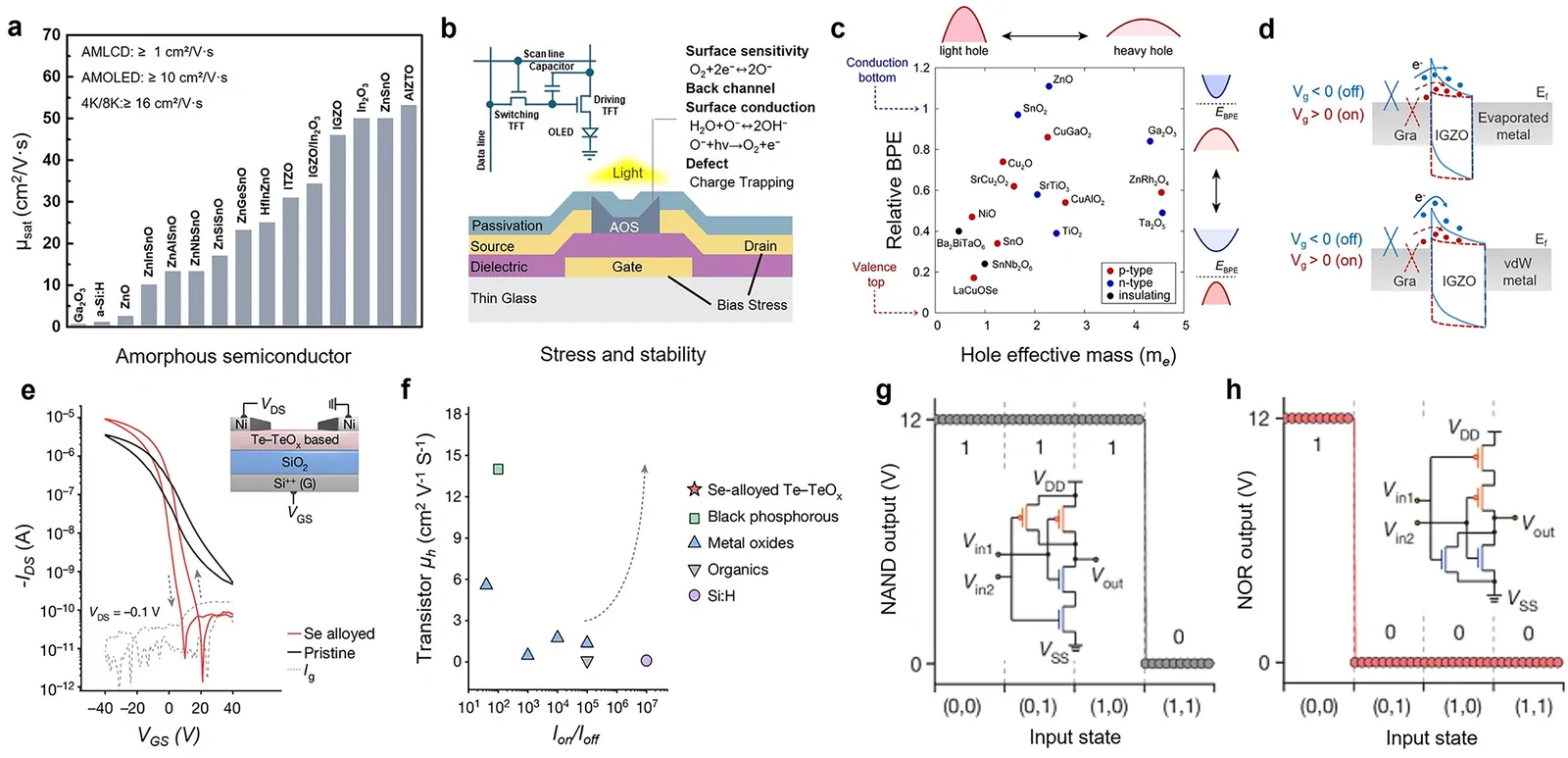
Transistors utilizing AOSs. **a** A summary of electron mobility (*μ*_e_) in n-type semiconductors using AOS. High *μ*_e_ improves device speed and resolution in display applications. **b** Schematic representation of stress generation in AOS transistors for display applications. **c** Graph summarizing material characteristics of *p* type, *n* type, and insulating states based on AOS band structure and hole effective mass. Reproduced with permission[203]. Copyright 2018, The Author(s). **d** Energy band diagram showing contact resistance reduction in InGaZnO_4_ channels using vdW contacts. Reproduced with permission [204]. Copyright 2024, The Author(s). **e** Transfer curves of *p* type AOS based on Te-TeO_*x*_ Se alloy, compared with pristine state. **f** Graph representing a specific performance metric for comparison of various material systems. Example of **g** NAND and **h** NOR gate output and circuit diagram using AOS transistors. Reproduced with permission [17]. Copyright 2024. The Author(s)

AOS materials offer distinct advantages over conventional silicon as transistor channel materials, particularly in the era of device scaling and heterogeneous integration. AOS materials exhibit intrinsically low leakage due to their wide bandgap, high carrier mobility even in amorphous phases, and superior compatibility with low-temperature, large-area fabrication techniques, unlike silicon, which suffers from increasing short-channel effects, high-leakage currents, and process-induced variability at nanometer dimensions [192]. Furthermore, their grain boundary-free structure ensures electrical uniformity, and dielectric interfaces enable reliable switching characteristics [193]. AOS materials have been extensively studied as a replacement for hydrogenated amorphous silicon (a-Si:H) in flat-panel display backplanes since Nomura et al. first demonstrated a flexible TFT using amorphous IGZO (a-IGZO) in 2004 [194]. The key performance metrics of AOS TFTs, including *µ*_0_, *V*th, and S.S., are influenced by device structure, fabrication process, material composition, gate dielectrics, and contact metal properties. Given their role in fast switching applications, high *μ*_FE_, a *V*th near zero, and a low S.S. are critical for optimal performance [195] As shown in Fig. 8a, a-Si:H TFTs have a low electron mobility (*μ*_e_) of approximately 1 cm^2^ V⁻^1^ s⁻^1^, whereas n-type AOS materials exhibit significantly higher mobility, offering superior electrical performance. In particular, a-IGZO’s high mobility makes it a key candidate for display backplanes. The growing demand for 4 K and 8 K resolution displays necessitates AOS-based switching matrix TFTs with enhanced mobility [196]. Figure 8a indicates that a minimum mobility of 16 cm^2^ V⁻^1^ s⁻^1^ is generally required for 4 K displays, highlighting AOS materials suitable for industrial adoption. Beyond high mobility, AOS supports low-temperature, large-area processing, making it well suited for flexible displays and wearable electronics, key applications for next-generation device technologies [192, 193, 195, 197–200].

AOS TFTs used in active matrix organic light emitting diode (AMOLED) and active matrix liquid crystal display (AMLCD) applications can suffer from instability due to gate bias stress, illumination, and environmental factors (Fig. 8b) [201, 202]. These reliability issues can be categorized into five key factors affecting long-term performance. The first factor is bias stress, which occurs due to charge trapping under prolonged gate voltage. Positive bias stress (PBS) causes electron trapping in the gate dielectric or at the channel/dielectric interface, shifting *V*th positively. Conversely, negative bias stress (NBS) leads to hole trapping or defect state formation, decreasing *V*th, making voltage stability a major challenge for display-driving devices. The second factor is illumination-induced instability, arising when AOS channels are exposed to high-energy light sources. While AOS materials have wide bandgaps and are stable under visible light, UV exposure generates photoinduced carriers, activating oxygen vacancies and interface traps, which degrade mobility and shift *V*th. Given their continuous operation in displays, minimizing photoinduced instabilities is critical. The third factor is ambient effects, caused by interactions with environmental gases like oxygen (O_2_) and moisture (H_2_O). Oxygen adsorption traps free electrons, reducing conductivity, while moisture alters hole concentration, leading to device instability. These effects are more pronounced in TFTs without passivation layers, making surface encapsulation essential for stability. The fourth factor is interface defects and charge trapping, which significantly affect AOS TFT stability at the gate dielectric interface. A high density of interface defects results in electron trapping, causing *V*th shifts (Δ*V*th) and increased S.S. over time. Gate dielectrics like SiO_2_ or Al_2_O_3_ play a crucial role in interface properties and long-term reliability, making dielectric optimization a key research area. The fifth factor is mechanical stress, especially relevant for flexible displays and wearable electronics [202]. AOS materials, being amorphous and grain boundary-free, offer uniform electrical properties compared to poly-Si. However, bending stress and mechanical deformation can increase interface defects, potentially affecting long-term durability. Research is focused on oxygen vacancy suppression through post-annealing, impurity doping, and surface passivation to enhance device stability.

Recent research has extended the use of AOS beyond displays to CMOS logic devices, where both *p* type and *n* type AOS are integrated to achieve low-power, high-performance transistors. While AOS has been widely used in displays due to its intrinsic *n* type characteristics and high *μ*_e_, realizing energy-efficient, high-speed logic circuits require both *p* type and *n* type transistors, similar to silicon-based CMOS technology. The key challenge is developing high-mobility *p* type AOS to balance circuit performance. Currently, *p* type AOS exhibits significantly lower hole mobility (*μ*_h_) than *μ*_e_ in *n* type AOS, leading to higher power consumption and slower switching speeds. Figure 8c illustrates the band structure and hole effective mass of various AOS materials, classifying them as *p* type, *n* type, or insulating. Most AOS materials naturally exhibit *n* type behavior, as electrons are less localized and move freely [203]. In contrast, *p* type AOS suffers from high hole effective mass and stronger hole localization, significantly limiting mobility. A key reason for low *μ*_h_ in p-type AOS is its metal–oxygen bonding, which traps holes around oxygen ions, restricting transport. Unlike conduction band electrons, valence band holes have a much higher effective mass, further reducing mobility. Additionally, oxygen vacancies in AOS act as electron donors, reinforcing *n* type conductivity. While suppressing oxygen vacancies is necessary for *p* type stabilization, it introduces fabrication challenges. Furthermore, most metal oxide semiconductors favor electron transport, making stable, efficient hole conduction rare. To overcome these limitations, several approaches have been explored. Alternative p-type oxides, such as Cu_2_O, NiO, and SnO, are under investigation, with SnO emerging as a promising candidate due to its higher *μ*_h_. Another approach is heterojunction structures, where p-type and n-type AOS are integrated into CMOS-compatible architectures. Transition metal doping (e.g., Cu, Cr, Fe) has also been explored to enhance *μ*_h_ by inducing p-type doping effects. Additionally, layered AOS structures, particularly HPs, are being studied for improved hole transport. If significant mobility improvements are achieved, AOS-based logic devices could become low-power alternatives to silicon-based semiconductors in select applications [203].

Beyond *μ*_h_, contact resistance is a critical challenge in AOS transistors, arising from Fermi-level pinning, low carrier concentration, interface defects, oxygen vacancies, metal reactivity, and structural inhomogeneity. To mitigate these issues, various techniques have been explored, including vdW metal contacts, oxygen vacancy control, and high-*K* dielectric integration. Figure 8d compares the energy band diagrams of evaporated metal contacts and vdW metal contacts on an IGZO channel. vdW contacts alleviate Fermi-level pinning, improving charge transport and reducing contact resistance. Figure 8d also illustrates that, in the on state, charge carriers (blue arrows) experience reduced scattering, enhancing electron transport efficiency [17]. In the off state, tunneling currents (red arrows) are reduced, minimizing leakage current. These findings confirm that vdW metal electrodes in vertical field-effect transistors (VFETs) exhibit lower contact resistance and superior on/off performance compared to conventional evaporated metal contacts, making them well suited for ultra-short-channel devices. Another promising strategy to reduce contact resistance is alloy doping, which modifies AOS conduction properties, enhancing charge transport and improving metal–semiconductor interface characteristics. Figure 8e presents the transfer characteristics of *p* type AOS based on Se-alloyed Te-TeO_*x*_, showing higher *I*_on_, lower *V*th, and improved stability compared to pristine AOS. Additionally, optimizing channel thickness and applying low-temperature annealing (~ 225 °C) have been shown to effectively lower contact resistance while maintaining device reliability. To assess the feasibility of AOS-based logic applications, Fig. 8f compares the *μ*_h_ and *I*_on_/*I*_off_ ratio across different semiconductor systems, confirming that AOS transistors outperform conventional metal oxides, organic semiconductors, and a-Si:H. Furthermore, Fig. 8g, h demonstrate the output characteristics of NAND and NOR gates, validating the feasibility of AOS-based logic circuits. Unlike traditional silicon-based CMOS processes, AOS transistors can be fabricated at lower processing temperatures, making them highly suitable for flexible electronics and large-area circuits [17]. Their compatibility with glass and plastic substrates enables the development of lightweight, low-power electronic systems, distinguishing them from conventional silicon-based ICs. The key semiconductor properties and transistor performance indicators of the emerging semiconductors are summarized in Table 2.

## Advanced Dielectrics

### High-*K*Dielectrics and 2D Material/High-*K* Integration

As semiconductor technology continues to advance, the steady reduction in transistor sizes, the nanoscale channel lengths, and the continuous thinning of gate dielectrics have introduced fundamental physical limitations in conventional CMOS transistors. These limitations include SCEs and direct tunneling leakage currents, which degrade device performance and increase power consumption. To address these challenges, two key strategies have been explored: reducing channel thickness to mitigate SCEs and suppressing tunneling leakage by replacing conventional SiO_2_ with high-*K* dielectrics [20, 86, 159]. The former approach utilizes 2D semiconductor materials, particularly vdW TMDs such as MoS_2_, WSe_2_, and BP [143, 206, 207]. These materials enable extreme thickness reduction down to the atomic level while maintaining improved gate control in MOSFET architectures. Additionally, their dangling bond-free surfaces help preserve high charge carrier mobility even at atomically thin dimensions. Beyond channel thickness scaling, replacing SiO_2_ with high-*K* dielectrics effectively suppresses tunneling leakage currents while maintaining sufficient physical thickness. The EOT is a critical parameter that represents the thickness of a high-*K* dielectric, enabling the same electrostatic effect as an equivalent SiO_2_ layer. Minimizing EOT is essential to transistor scaling. To reduce direct tunneling leakage and enhance energy efficiency, FinFET manufacturing has explored high-*K* dielectrics such as Al_2_O_3_, HfO_2_, Si_3_N_4_, ZrO_2_, TiO_2_, Y_2_O_3_, Ta_2_O_5_, and LaZrO_2_ [208]. Intel’s 14 nm FinFET technology, for example, integrates a 2.6 nm HfO_2_ gate dielectric, achieving an EOT of 0.9 nm. In low-standby-power CMOS applications, it maintains a gate leakage current (*I*_g_) of 1.5 × 10⁻^2^ A cm⁻^2^ and an interface state density of approximately 10^10^ cm⁻^2^ eV⁻^1^, setting a significant performance benchmark for emerging transistor technologies [159, 209]. These advancements underscore the necessity of continuous innovation in high-*K* dielectric integration to enable next-generation semiconductor devices.

**Fig. 9:**
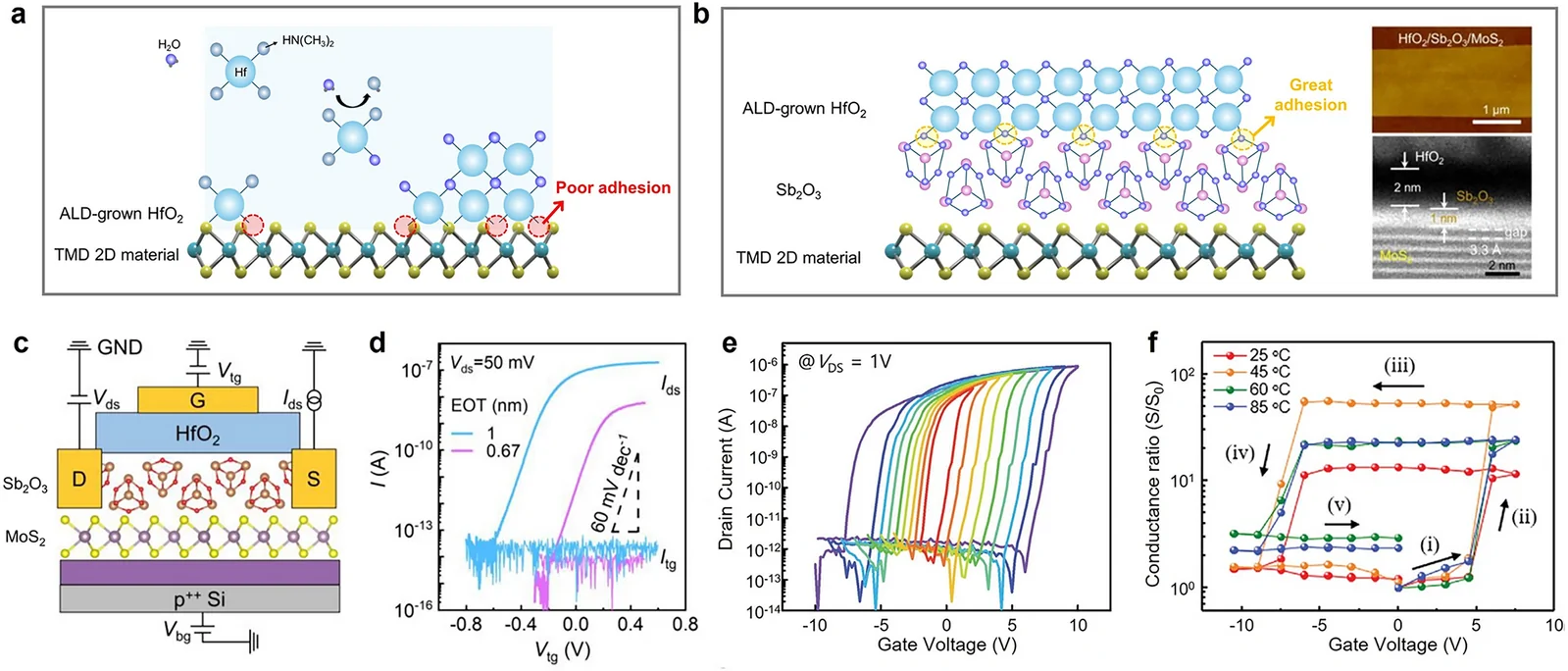
High-*K* & ferroelectric usage in recent FETs. **a** Illustration of poor HfO_2_ growth via ALD on monolayer 2D TMD materials. **b** Improved quality of ALD-grown HfO_2_ with the insertion of a 1-nm-thick Sb_2_O_3_ buffer layer between the 2D material and the high-*K* dielectric layer. **c** Schematic of a dual-gated MoS_2_ FET employing Sb_2_O_3_/HfO_2_ bilayer gate dielectrics. **d** Transfer characteristics of the two dual-gated MoS_2_ devices with two types of Sb_2_O_3_/HfO_2_ gate dielectrics that show an EOT of 1 nm and 0.67 nm. Reproduced with permission [216], Copyright 2023, Elsevier. **e** Gate sweep-dependent transfer behavior of α-In_2_Se_3_ FeFET with hBN/CIPS. Reproduced with permission [18]. Copyright 2022, Wiley–VCH GmbH. **f** Nonvolatile modulation of channel conductance after application of gate voltage pulses. Reproduced with permission [19]. Copyright 2022, Wiley–VCH GmbH

Recent advancements in high-*K* dielectrics emphasize not only achieving a high-*K* but also meeting essential criteria such as low interfacial state density, minimal dielectric loss (tan δ < 0.01), a wide bandgap for effective suppression of leakage currents, and compatibility with existing semiconductor fabrication processes [210–212]. These requirements are particularly crucial for the successful integration of high-quality dielectrics with 2D semiconductors, which possess vdW surfaces that pose challenges for uniform dielectric deposition. According to the international roadmap for devices and systems (IRDS), state-of-the-art MOSFETs at the 5 nm technology node require an EOT below 1 nm and a *V*_dd_ of less than 0.8 V. However, the inert, dangling bond-free surface of 2D materials presents significant challenges for uniform and scalable deposition of ultra-thin dielectrics (Fig. 9a) [20]. To address these challenges, the dielectric deposition process must be nondestructive and ideally form a vdW interface with the 2D semiconductor to preserve its intrinsic properties while minimizing interfacial state density. Various approaches have been explored, including direct deposition, oxidation of deposited metals, surface pretreatment, and organic seeding layers. Despite these efforts, a universally applicable method for integrating high-quality dielectric layers with EOTs below 1 nm on 2D materials has yet to be established. Consequently, although 2D FETs are expected to exhibit superior gate electrostatics compared to conventional semiconductors, they still require higher operating voltages than the latest Si-based MOSFETs, posing a fundamental limitation to their widespread adoption.

To overcome this limitation, Xu et al*.* proposed a novel approach for integrating an ultra-thin high-*K* dielectric layer (EOT < 1 nm) onto 2D materials. In their work, antimony trioxide (Sb_2_O_3_) was employed as a buffer layer interfacing with 2D semiconductors through van der Waals interactions, enabling the realization of an oxide–semiconductor interface with minimal interfacial trap density. Additionally, Sb_2_O_3_ provides a hydrophilic surface, enabling uniform deposition of ultra-thin high-*K* dielectric layers (HfO_2_, Al_2_O_3_, ZrO_2_) via standard atomic layer deposition (ALD) even on hydrophobic 2D materials [20]. Using this method, a hybrid inorganic dielectric layer with an EOT of 0.67 nm was successfully formed, marking the thinnest EOT reported for 2D materials to date (Fig. 9b) [20]. Figure 9c illustrates a monolayer MoS_2_ FET with the Sb_2_O_3_/HfO_2_ hybrid dielectric, comparing devices with 3 nm and 5 nm dielectric thicknesses (EOT 0.67 and 1.01 nm). The thinner dielectric enhances electrostatic control, leading to a sharp *I*_ds_ transition around 0.4 V, indicating high gate efficiency. Figure 9d shows that the devices achieve S.S. values near 60 mV dec⁻^1^, demonstrating low leakage current and effective gate control. Statistical analysis confirms consistent S.S. values (60–75 mV dec⁻^1^), reinforcing the hybrid dielectric’s reliability in minimizing power consumption and enhancing transistor performance. Additionally, a thinner EOT reduces the *V*th, enabling operation at a lower *V*_dd_, which helps decrease power consumption. In contrast, a thicker EOT (1 nm) results in weaker gate control, leading to an increased S.S. value and slower switching speed [20]. Similarly, Li et al*.* demonstrated an alternative approach using perylene-tetracarboxylic dianhydride (PTCDA) molecular crystals as a seeding layer for ALD of high-*K* dielectrics [159]. The thickness of the molecular crystal could be precisely controlled to a monolayer (~ 0.3 nm) via self-limited epitaxy, allowing for high-*K* dielectric integration on graphene and MoS_2_. The PTCDA/HfO_2_ gate dielectric exhibited a low leakage current (< 10⁻^2^ A cm⁻^2^) and a high-breakdown electric field (*E*ₒₖ = 16.5 MV cm⁻^1^), meeting the ITRS standards for low-power applications. Furthermore, MoS_2_ and WSe_2_ CMOS transistors demonstrated a *V*_dd_ comparable to state-of-the-art Si CMOS while maintaining high *I*_on_/*I*_off_ ratios and achieving S.S. values near the thermionic limit of 60 mV dec⁻^1^. Both the Sb_2_O_3_ buffer layer and the perylene-3,4,9,10-tetracarboxylic dianhydride (PTCDA) molecular seeding layer effectively address the challenges of depositing dielectric layers on the inert, dangling bond-free surface of 2D semiconductors. By reducing EOT below 1 nm, they enable low S.S. (~ 60 mV dec⁻^1^) and high *I*_on_/*I*_off_ current ratios, making them promising strategies in the development of next-generation low-power transistors [159].

### Ferroelectric Materials

The ferroelectric dielectric layer is a key component of the ferroelectric field-effect transistor (FeFET), enabling spontaneous polarization retention under an external electric field, unlike conventional insulating layers. This characteristic allows FeFETs to function as nonvolatile memory devices, operating at lower power consumption while achieving fast read/write speeds compared to traditional DRAM or flash memory [213]. Additionally, the continuous polarization variation in the ferroelectric dielectric layer enables multi-level data storage rather than binary storage, enhancing its potential for high-density memory applications [19].

Ferroelectric dielectric layers can be integrated with various semiconductor materials. The most common ferroelectric insulators include hafnium oxide-based ferroelectrics and conventional perovskite ferroelectrics such as Pb(Zr,Ti)O_3_ (PZT) [213]. While PZT exhibits excellent ferroelectric properties, its high processing temperature and environmental concerns, due to lead content, limit its compatibility with CMOS technology. Conversely, Hf_0.5_Zr_0.5_O_2_ (HZO) is a promising alternative as it retains ferroelectricity even at relatively low fabrication temperatures and is compatible with existing semiconductor manufacturing processes [80, 214]. However, HZO also requires stringent thermal and chemical conditions to stabilize its orthorhombic phase, and its relatively lower *K* and charge trapping issues present significant challenges. To overcome these limitations, 2D ferroelectric materials have gained increasing attention. Unlike conventional ferroelectrics, 2D ferroelectrics maintain their polarization at atomic thicknesses, offering advantages such as high scalability, nonvolatile memory functionality, and applicability in neuromorphic computing. Representative 2D ferroelectric materials include α-In_2_Se_3_, SnS, and CuInP_2_S_6_ (CIPS), which exhibit directional ferroelectricity while demonstrating enhanced stability and lower power consumption compared to conventional ferroelectrics [213]. Particularly, α-In_2_Se_3_ is a promising candidate for nonvolatile memory and neuromorphic applications due to its robust polarization retention at ultra-thin thicknesses. Ferroelectric dielectric layers can also be integrated with various semiconductor materials to impart multi-functionality to FeFETs. A notable example is the VO_2_-based ferroelectric transistor, which leverages a heterostructure combining BiFeO_3_ (BFO) with VO_2_ [18]. Figure 9e illustrates the transfer characteristics of this device, showing the conductance ratio (*S*/*S*_0_) as a function of *V*_g_ across multiple temperatures (25, 45, 60, and 85 °C). VO_2_, known as a prototypical phase transition compound, exhibits a metal–insulator transition (MIT) at around 68 °C. The device exhibits a distinct hysteresis loop, indicating that the MIT in VO_2_ can be non-volatilely modulated through ferroelectric gating. As the gate voltage increases (i → ii), a sharp rise in conductance occurs, reflecting an electric field-induced MIT from the insulating to the metallic state. Conversely, as the gate voltage decreases (iii → iv), VO_2_ transitions back to its insulating state, forming a hysteresis loop. The hysteresis width and *V*th of the transition are temperature-dependent, attributed to the interplay between polarization at the VO_2_/ferroelectric interface and charge injection effects. Notably, the decrease in the MIT *V*th at higher temperatures suggests a reduction in the transition energy barrier, facilitating enhanced charge transport. These findings confirm that ferroelectric gating enables nonvolatile control of MIT in VO_2_, demonstrating its potential for low-power memory and neuromorphic computing applications.

Despite their promise, conventional FeFETs face limitations such as short retention time, depolarization fields, and *I*_g_, which hinder their commercialization. These issues arise from potential drops at the ferroelectric/semiconductor interface and charge trapping effects, leading to *V*th drift and instability in memory states. To address these challenges, ferroelectric semiconductor field-effect transistors (FeS-FETs) have been proposed. Unlike traditional FeFETs, FeS-FETs utilize a ferroelectric semiconductor as the channel material rather than a ferroelectric insulator. This structural difference enables FeS-FETs to form two nonvolatile polarization states within the semiconductor itself, effectively mitigating depolarization field effects and significantly improving retention time and endurance compared to FeFETs. Furthermore, to overcome the inherent limitations of both FeFETs and FeS-FETs, a new Fe_2_-FET architecture has been introduced. Fe_2_-FETs integrate both ferroelectric insulators and ferroelectric semiconductors, effectively neutralizing the counterclockwise hysteresis of FeFETs and the clockwise hysteresis of FeS-FETs to achieve hysteresis-free switching characteristics. By precisely engineering the polarization charge at the ferroelectric insulator/ferroelectric semiconductor interface, net polarization can be minimized, thereby eliminating hysteresis and improving switching efficiency [215]. Recent research has explored van der Waals heterostructures, incorporating layered ferroelectrics such as CIPS and α-In_2_Se_3_ into FeFETs to overcome the limitations of ferroelectric oxides, achieving wider memory windows and enhanced polarization retention (Fig. 9f) [19]. Unlike conventional FeFETs, where the ferroelectric insulator indirectly modulates the electron density in the channel, FeS-FETs directly leverage the intrinsic ferroelectric properties of the semiconductor channel, enabling superior channel control. These advances contribute to enhancing the durability and reliability of ferroelectric dielectric layers, further extending their applicability in neuromorphic computing and high-performance memory devices. The development of Fe₂-FETs and next-generation hybrid ferroelectric devices has the potential to maximize the performance of ferroelectric transistors, integrating them seamlessly with existing semiconductor technologies to accelerate the commercialization of ultra-low-power, high-performance electronic devices.

### 2D Dielectrics

Dielectric materials play a crucial role in enabling next-generation nanoelectronics, providing electrostatic control, charge insulation, and interface stability for high-performance FETs, quantum devices, and neuromorphic systems. Unlike conventional bulk dielectrics, 2D dielectrics offer atomically smooth surfaces, minimal charge trapping, and strong compatibility with van der Waals heterostructures, making them highly attractive for miniaturized and high-efficiency electronics. The dangling bond-free structure of 2D dielectrics enables the formation of pristine interfaces in 2D/2D vdW heterostructures, significantly reducing Coulomb impurity scattering and interface-induced charge trapping [113, 217–219]. This clean interface not only enhances carrier mobility but also improves device stability by minimizing defects at the dielectric/channel interface, which is critical for achieving high-performance FETs, ultra-low-power electronics, and quantum-coherent systems. These materials can be broadly classified into low-*K* and high-*K* dielectrics based on their dielectric constant, where low-*K* materials mitigate parasitic capacitance and charge scattering, while high-*K* materials enhance gate control and suppress leakage currents. In particular, the integration of high-*K* 2D dielectrics into vdW heterostructures provides an effective means of enhancing electrostatic gating while preserving the intrinsic properties of atomically thin semiconductors, thereby enabling high-speed and energy-efficient nanoelectronics.

**Fig. 10:**
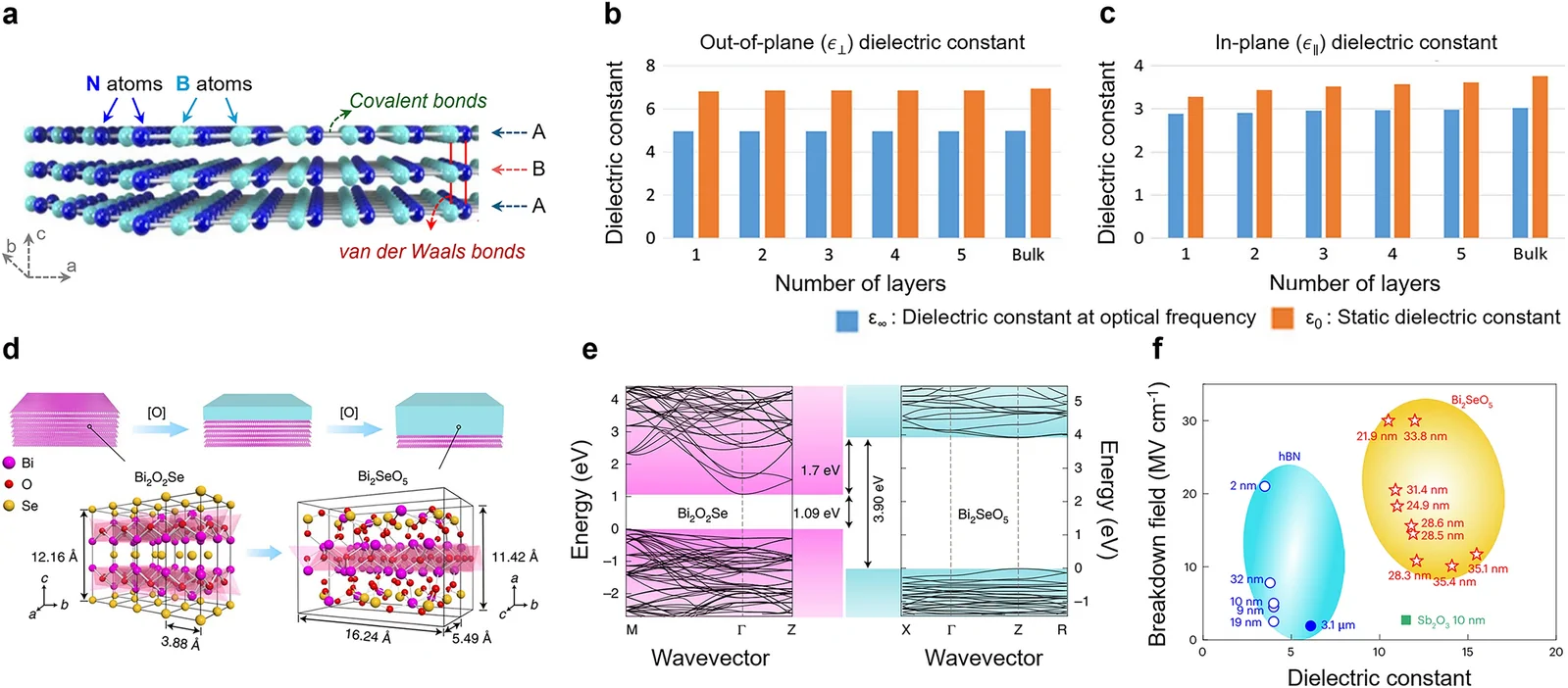
Properties and applications of hBN and 2D high-*K* dielectric materials. **a** Crystal structure of hBN and dielectric constant variations in hBN layers: **b** out-of-plane (ϵ_⊥_) and **c** in-plane (ϵ_∥_) dielectric constants as a function of the number of layers. Reproduced with permission from [220]. Copyright 2018, Springer Nature. **d** Schematic diagram of the oxidation process for fabricating 2D high-*K* dielectric material Bi_2_SeO_5_ from Bi_2_O_2_Se, demonstrating the structural evolution upon oxidation. **e** Band structure comparison between Bi_2_O_2_Se and Bi_2_SeO_5_, showcasing the changes in energy levels, including the bandgap widening and electronic properties. Reproduced with permission from [225]. Copyright 2020, Springer Nature. **f** Comparative plot of dielectric constant versus breakdown field for 2D material. Reproduced with permission from [230]. Copyright 2023, Springer Nature

hBN [220–224] is the most widely utilized low-*K* 2D dielectric due to its wide bandgap, excellent chemical and thermal stability, and strong insulating properties. Its atomic structure, consisting of alternating boron (B) and nitrogen (N) atoms covalently bonded within layers and interacting weakly via van der Waals forces (Fig. 10a), facilitates seamless integration into van der Waals heterostructures. The anisotropic dielectric properties of hBN are presented in Fig. 10b, c, showing a higher out-of-plane dielectric constant (ϵ_⊥_ ≈ 5–7) compared to the lower in-plane dielectric constant (ϵ_∥_ ≈ 3–4) [220]. This dielectric anisotropy makes hBN ideal for minimizing charge impurity scattering while maintaining sufficient electrostatic control. The hBN is primarily synthesized via CVD and mechanical exfoliation, providing high-crystallinity films suitable for FET applications. For device integration, hBN is transferred onto silicon, glass, or flexible polymer substrates using polymer-assisted techniques, preserving its atomic smoothness and minimizing contamination. A functional derivative, hexagonal boron nitride oxide (h-BNO), is obtained through controlled oxidation, which introduces oxygen functionalities into the boron nitride lattice via selective nitrogen replacement or covalent B–O bond formation. This chemical modification enhances h-BNO’s dielectric strength and thermal resistance while preserving its crystalline integrity, making it a promising candidate for high-frequency electronics and flexible devices.

While hBN serves as a reliable low-*K* dielectric, high-*K* dielectrics are essential for improving gate efficiency, reducing subthreshold leakage, and enabling ultra-scaled transistors. High-*K* 2D oxides such as Bi_2_O_2_Se and its oxidized derivatives Bi_2_SeO_5_ [225–229] exhibit superior dielectric performance. As shown in Fig. 10d, controlled oxidation of Bi_2_O_2_Se leads to the formation of Bi_2_SeO_5_, which possesses an increased dielectric constant and a wider bandgap. Electronic structure analysis (Fig. 10e) indicates that Bi_2_SeO_5_ has a bandgap of 3.9 eV, significantly larger than that of Bi_2_O_2_Se (1.7 eV), improving its suitability as a high-*K* gate dielectric. The breakdown field versus dielectric constant relationship (Fig. 10f) further highlights Bi_2_SeO_5_’s enhanced dielectric properties, positioning it as a strong candidate for next-generation gate oxides. These characteristics enable improved gate efficiency, reduced subthreshold leakage, and enhanced electrostatic control in ultra-scaled transistors.

The integration of low-*K* and high-*K* 2D dielectrics is crucial for advancing nanoelectronic devices. hBN remains the most reliable low-*K* dielectric for passivation, encapsulation, and interfacial engineering due to its ultra-smooth surface and chemical stability. Meanwhile, high-*K* materials such as Bi_2_SeO_5_ provide enhanced gate control and reduced leakage currents, addressing key limitations in transistor scaling. Functionalized dielectrics like h-BNO further expand the potential applications of 2D dielectrics in extreme operating conditions and flexible electronics. Future research should prioritize scalable synthesis methods, interfacial defect minimization, and optimized integration strategies to fully exploit the advantages of 2D dielectrics in next-generation electronic and optoelectronic applications.

## Future Interconnects

### Scaling Interconnections in BEOL

**Fig. 11:**
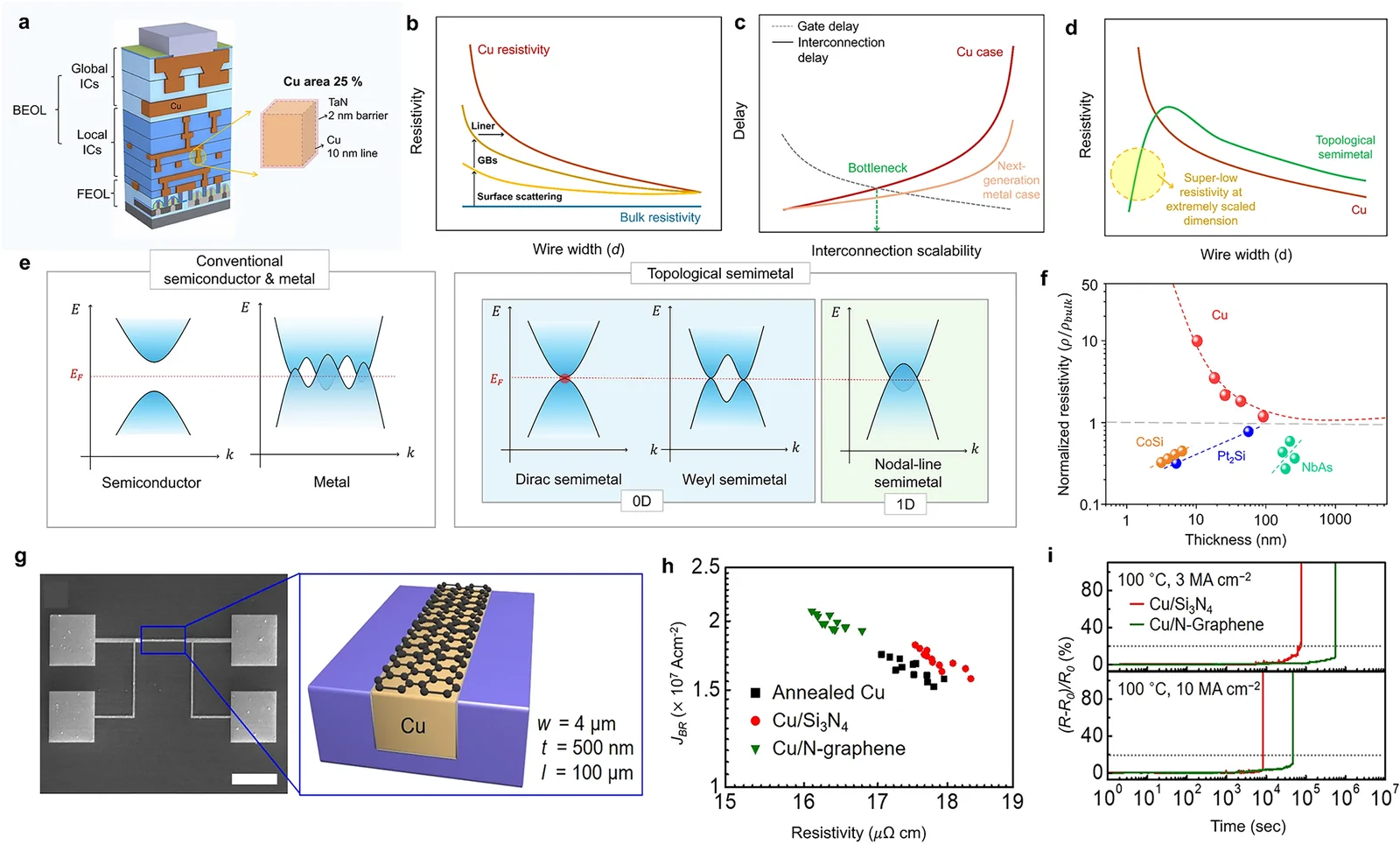
Challenges and advancements in next-generation interconnection materials beyond Cu. **a** Schematic representation of BEOL interconnects, highlighting the Cu area as 25% and integration of TaN for enhanced reliability. **b** Resistivity trend depending on the Cu wire width, showing some factors to increase resistivity at the extremely scaled dimension: surface scattering, grain boundaries, and line processing. **c** Growing gap between the gate delay and interconnection delay as the interconnection scalability advances. The adoption of next-generation metals as alternative interconnect materials can mitigate the bottlenecks that hinder further scalability improvements. **d** Comparative analysis of the conventional Cu and topological materials as a function of wire width, illustrating the superior resistivity of topological semimetals due to their enhanced electron transport properties at extremely reduced dimensions. **e** Energy band structures of conventional semiconductor, metal, and topological semimetals. Various topological semimetals are categorized based on the dimensionality of their band crossing points as 0D and 1D. **f** Resistivity trends of representative topological semimetals compared to conventional Cu under varying thicknesses. Data for these plots were referred from recent studies [103, 239, 240]. **g** SEM image of a fabricated interconnect structure alongside an enlarged 3D schematic of an N-doped graphene-capped Cu interconnect within a damascene-patterned structure. **h** Plot of breakdown current density versus resistivity for annealed Cu, Si_3_N_4_-capped Cu, and N-doped-graphene-capped Cu, showing the enhanced conduction reliability of graphene-capped case. **i** Breakdown endurance of interconnects comparing Si_3_N_4_-capped Cu and N-doped-graphene-capped Cu under 100 °C and constant current density. Reproduced with permission from [23], Copyright 2021, Nature Publishing Group

Since the introduction of dual-damascene processing in the back-end-of-line (BEOL) structure, copper (Cu) has been the dominant interconnect material, first proposed by IBM [231]. However, the inherent challenges of Cu, such as electromigration and diffusion, necessitate the integration of barriers and liner materials, including TaN and Co layers, to mitigate these issues. As the size of transistors decreases, interconnect dimensions have also scaled down to nanometer widths, connecting billions of transistors in an integrated circuit. However, the physical limitations of Cu interconnects are becoming increasingly evident as device scaling approaches its fundamental limits. In dual-damascene process for the BEOL fabrication, the fraction of Cu occupies only 25% among the total interconnection part, as TaN and Co layers are required to prevent electromigration and diffusion (Fig. 11a). This dimensional scaling issue exacerbates RC (resistance–capacitance) delay, leading to excessive power consumption in advanced semiconductor nodes [232]. As Cu interconnect dimensions approach the nanoscale, resistivity increases significantly due to enhanced surface scattering and grain boundary effects. This rise follows the Fuchs–Sondheimer (FS) and Mayadas–Shatzkes (MS) models, where electron mean free path is limited by surface roughness and grain boundary reflections [233]. As a result, the effective conductivity of Cu interconnects degrades, leading to severe RC delay and power dissipation in advanced technology nodes. Figure 11b illustrates the exponential resistivity increase at the sub-10 nm regime, leading to severe interconnection delay issues.

To mitigate the increase of resistivity in Cu interconnects, recent industry efforts have explored alternative liner and barrier materials such as Ru and Co [234]. These materials have shown improved electromigration resistance and reduced grain boundary scattering, leading to lower resistivity compared to conventional Cu-based interconnects [235, 236]. Despite these advancements, Ru and Co still exhibit higher bulk resistivity (~ 10 μΩ cm) than Cu (~ 1.7 μΩ cm), and their integration into BEOL remains challenging due to deposition complexity and cost [236]. Consequently, Cu interconnects face a technical bottleneck in scaling (Fig. 11c), necessitating the development of alternative interconnect materials. Given the increasing limitations of Cu interconnects, topological semimetals (TSMs) have emerged as promising alternatives due to their unique scattering-free surface conduction properties Topological semimetals such as TaAs, NbAs, and MoP, unlike Cu possess protected surface states that facilitate electron transport without backscattering, dramatically reducing resistivity. These materials exhibit extremely low resistivity at the sub-10 nm scale (Fig. 11d), addressing the interconnection delay issue. A notable example is TaAs nanobelt structures (~ 200 nm width), achieving ultra-low resistivity (~ 35 μΩ cm), significantly outperforming Cu in nanoscale dimensions. Charge carriers in TSMs are less affected by grain boundaries and surface scattering, due to the distinguished energy band structures in topological semimetals, where the crossing points between valence band and conduction band are formed with Weyl semimetal. The feasibility of Weyl semimetal interconnects in standard CMOS-compatible BEOL processing is an active research area. Process challenges such as patterning, deposition techniques, and material compatibility must be addressed before widespread adoption.

1D metallic nanowires and 2D layered metals could complement or replace conventional Cu-based interconnects [237, 238]. Recent advances highlight mirror twin boundaries in TMDs, as promising interconnect materials. These dislocation-free structures enable quasi-ballistic transport, reducing electron scattering and enhancing current-carrying capacity compared to Cu [98]. The low resistivity at nanoscale dimensions positions them as promising candidates for overcoming interconnect scaling challenges. These novel materials offer higher carrier mobility and reduced electron scattering, making them potential candidates for sub-3 nm technology nodes and beyond.

### Advancing Beyond Ti/Au Contacts

The continued miniaturization of semiconductor devices necessitates advancements in interconnect materials to address scalability, reliability, and electrical performance challenges. While Cu/TiN-based electrodes have been widely utilized due to their low resistivity and process compatibility not to induce Cu interdiffusion, a high contact resistance induced by Schottky barrier has not been completely resolved to actively integrate in sub-10 nm technology node device [241]. Many approaches have been explored to reduce contact resistance at metal–silicon interfaces, particularly through the insertion of metal–silicon alloys—commonly known as silicides such as NiSi, CoSi_2_, or TiSi_2_ [242, 243]. However, these methods primarily address contact resistance in conventional silicon-based devices. In the case of low-dimensional semiconductors, where the intrinsic tunneling barrier at the metal/semiconductor interface dominates the contact resistance, alternative metals such as bismuth (Bi) or molybdenum (Mo) [244, 245] should be considered to mitigate this fundamental limitation. Mo has demonstrated strong adhesion to semiconductor surfaces and forms a lower Schottky barrier compared to conventional Ti/Au contacts [246]. Experimental studies have shown that Mo/TiN-based contacts can achieve an order-of-magnitude reduction in contact resistance, making them a promising candidate for next-generation semiconductor applications [247]. For MoS_2_ transistor, Mo electrodes could be a promising alternative for optimizing device performance. Yoo et al*.* demonstrated that Mo-MoS_2_ edge contacts effectively mitigate performance degradation associated with increasing channel thickness, offering a viable strategy for enhancing device scalability [248]. Moreover, Mo is inherently compatible with CMOS fabrication processes, offering seamless integration into industrial-scale manufacturing [247]. Similarly, Bi has attracted attention as an interfacial material due to its low work function, which effectively reduces the Schottky barrier height, facilitating improved charge injection. Shen et al*.*, demonstrated that Bi deposition on monolayer MoS_2_-based FETs significantly reduced contact resistance, achieving an ultra-low RC of 123 Ωμm at a carrier density of 1.5 × 10^13^ cm⁻^2^ [160]. These findings highlight the potential of Mo and Bi as viable solutions for overcoming the limitations of traditional Ti/Au contacts while ensuring compatibility with existing semiconductor processing technologies.

Beyond Mo and Bi-based adhesion layers, Weyl semimetals have gained interest as a disruptive alternative due to their unique topological electronic properties. Unlike conventional metals, WSMs exhibit topologically protected surface states that allow nearly ballistic charge transport, effectively eliminating scattering-related resistive losses [249]. This intrinsic characteristic makes WSMs highly advantageous for scaled transistor technologies where reducing contact resistance and maintaining high carrier mobility are crucial. Furthermore, WSMs lack a conventional bandgap, enabling them to form Ohmic contacts with semiconductors without the need for additional interface engineering. At this point, WSMs exhibit a distinct band structure, where conduction and valence bands touch at discrete Weyl nodes, unlike conventional semiconductors with bandgaps or metals with overlapping bands (Fig. 11e). Studies on NbAs-based electrodes have revealed that surface conduction states can contribute up to 70% of the total conductivity, further enhancing charge transport efficiency in scaled devices [102]. The combination of high carrier mobility and suppressed electron backscattering positions WSMs as a strong contender for the next generation of interconnect materials [250]. To highlight the resistivity advantage of WSMs at sub-10 nm interconnect dimensions, they maintain low resistivity at reduced thickness unlike Cu (Fig. 11f). Recently, Rocchino fabricated the transistor based on NbP with strong manetoresistive coupling [251]. The resistance of NbP was modulated using a locally induced magnetic field generated by a superconducting gate.

**Table 2:** Comparative summary of the bandgap, carrier mobility, thickness, and *I*_on_/*I*_off_ ratio of emerging channel materials for next-generation transistors. All data are extracted from values described in the respective sections [16, 141, 143, 144, 147–149, 190, 205]

Channel materials	Bandgap (eV)	Mobility (cm^2^/Vs)	Thickness (nm)	*I*_on_/*I*_off_ ratio
2D vdW	~ 0.3 eV (BP bulk) ~ 2.0 eV (BP monolayer)	MoS_2_: 80 (FET), 133 (Hall) InSe: ~ 9100 (Hall) WSe_2_: 27 (FET)	mono: < 1 InSe: ~ 9	MoS_2_: ~ 10^5^ WSe_2_: > 10^7^ (FET)
Halide Perovskite	–	CsFAPEA: 70 (FET)	–	CsFAPEA: 10^8^ (FET)
Mott Insulator (VO_2_)	Insulating (M1) to metallic (R) phase transition	–	~ 6 (Nb-TiO_2_/VO_2_)	~ 10^3^ (MIT switch)
Amorphous Oxide	~ 3.0	~ 10–50 (FET)	–	~ 10^9^ (FET)

Despite their promising electrical properties, the practical integration of WSMs into CMOS-compatible BEOL processes remains an ongoing research challenge. Key barriers include the scalable synthesis of high-quality WSM thin films, precise patterning, and deposition techniques that align with standard semiconductor processing methods. Current research efforts are exploring ALD and CVD techniques to facilitate the growth of WSMs with controlled thickness and uniformity for interconnect applications. Additionally, advancements in etching methodologies and material compatibility assessments are required to ensure seamless integration into current semiconductor manufacturing workflows. While these challenges must be addressed, the potential of WSMs to eliminate contact resistance constraints makes them an attractive candidate for next-generation interconnect technology. Graphene-capped Cu interconnects have been explored as another promising alternative, offering low resistivity, high thermal stability, and improved electromigration resistance (Fig. 11g). Graphene-capped Cu interconnects have superior conduction reliability with extended failure times of 3 and 10 MA cm⁻^2^ (Fig. 11h, i) [23]. As reliability is a key requirement for industrial application, materials that exhibit both enhanced endurance and intrinsic CMOS process compatibility are particularly promising. CMOS process compatibility is a fundamental requirement for the commercialization of new interconnect material (Table 3). Mo is already utilized in semiconductor processing and has demonstrated its feasibility for industrial-scale applications, while Bi, though less commonly integrated, has shown promising results in experimental settings and could be further optimized for widespread adoption. On the other hand, Weyl semimetals, though disruptive in nature, require further advancements in process scalability, interface engineering, and industrial integration before they can replace conventional interconnect materials. As semiconductor scaling approaches its fundamental limits, Ti/Au-based contacts face increasing challenges due to their high contact resistance, Schottky barrier formation, and limited thermal stability. To address these issues, Mo and Bi-based adhesion layers provide a CMOS-compatible, low-resistance solution, while Weyl semimetals offer a novel, barrier-free alternative for ultra-scaled transistors. Future research should focus on refining scalable deposition techniques, optimizing interfacial properties, and developing integration strategies that will enable these advanced interconnect materials to bridge the gap between current BEOL limitations and the demands of next-generation semiconductor devices.

**Table 3:** Next-generation interconnection materials and their conduction type, advantageous microstructure, and potential for interconnection scaling. The data for these table were referred from recent studies [23, 252]

Dimensional metallic materials	Conduction	Microstructure	Dimensional anisotropy	Potential
3D metals—elements	Co, Mo, Ru	Isotropic	Polycrystalline	State of art
3D metals—compounds	NiAl, Al_3_Sc			Improved scaling
2D metals	Cr_2_AlC, PtCoO_2_	2D anisotropic	Textured	Less surface scattering
2D materials	Graphene	2D anisotropic	Textured	Less surface scattering
1D metals	CoSn, CoSi	1D anisotropic	Epitaxial	Suppression of surface scattering
Weyl semimetals	NbAs, MoP, Pt_2_Si	Surface state, anisotropic	Epitaxial	Perimeter scaling

## Reliability and Device Lifetime

As the scaling of MOSFETs approaches its physical and quantum mechanical limits, ensuring long-term device reliability has become a critical concern for sustaining performance and operational stability in modern electronics. In advanced semiconductor nodes, even minor reliability issues can severely impact circuit functionality, leading to increased power consumption, performance degradation, and early device failure. This is particularly crucial for applications requiring ultra-low power operation, high-speed processing, and long-term durability, such as AI, edge computing, and emerging quantum computing technologies.

**Fig. 12:**
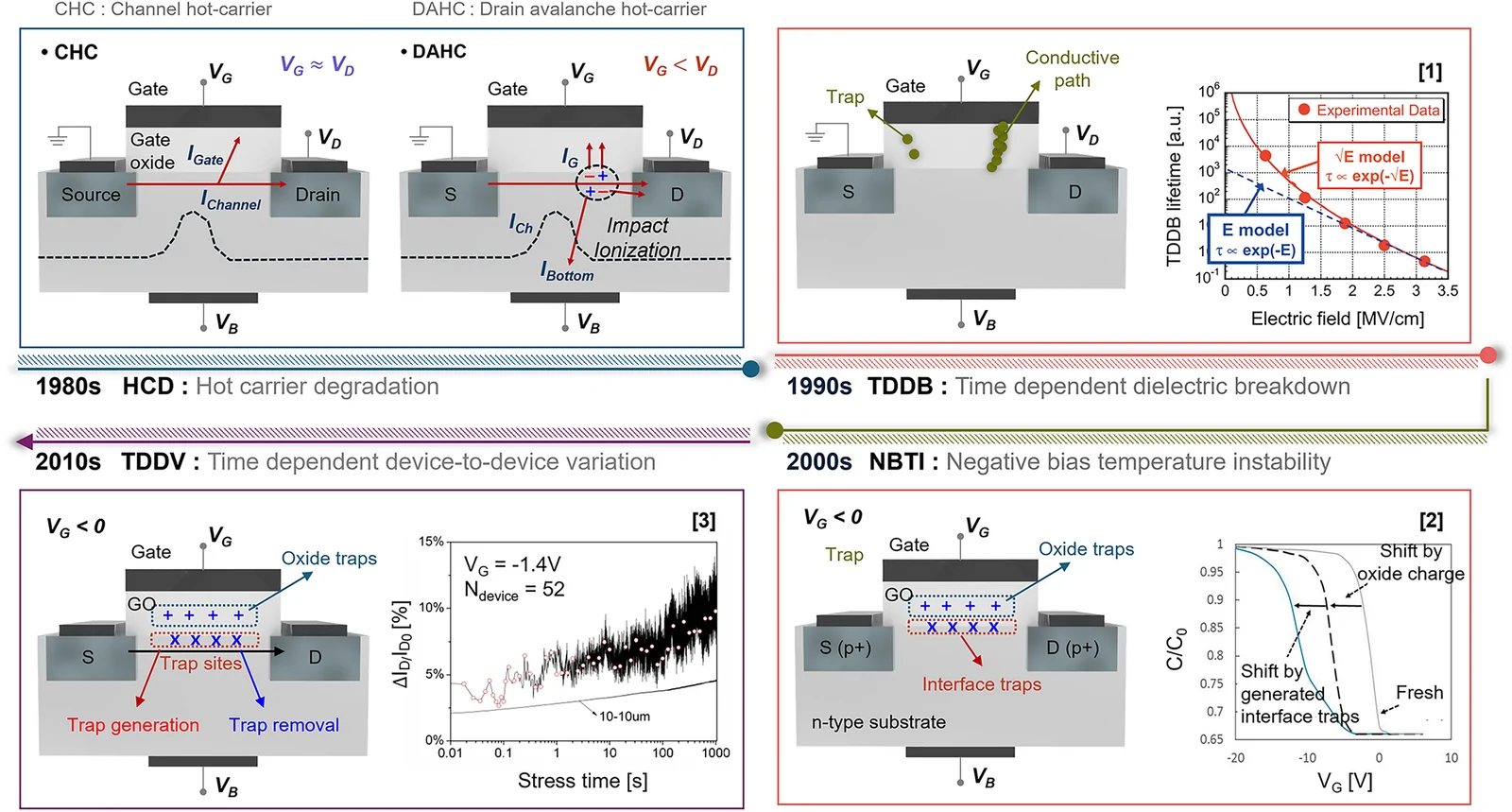
Overview of reliability issues in MOSFET devices, highlighting key mechanisms and degradation processes. CHC and DAHC effects: Depiction of impact ionization leading to carrier injection (gate leakage current *I*_g_) and channel degradation under varying gate-to-drain voltage conditions. TDDB: Formation of conductive paths in the oxide layer due to electric field-induced stress. NBTI: Shifts in device characteristics due to interface and oxide trap generation. TDDV: Impact of oxide trap generation and removal on statistical device performance, with measured variability under negative gate bias. Reproduced with permission from [274]. Copyright 2013, Elsevier. Reproduced with permission [275]. Copyright 2017, Elsevier. Reproduced with permission [259]. CC.BY.3.0

Various degradation mechanisms (Fig. 12), including hot-carrier degradation (HCD) [253, 254], time-dependent dielectric breakdown (TDDB) [255], negative bias temperature instability (NBTI) [256–258], and time-dependent device-to-device variation (TDDV) [259], impose significant constraints on device performance, reliability, and lifetime. These reliability challenges are exacerbated as transistor dimensions shrink to the sub-3 nm regime, where increased electric fields, enhanced quantum effects, and interfacial defects contribute to accelerated degradation. A comprehensive understanding of these reliability challenges and developing strategies to mitigate their impact will be fundamental in enabling the next wave of semiconductor technologies.

### Hot-Carrier Degradation and Drain Avalanche Hot-Carrier Effects

Hot-carrier degradation (HCD) occurs due to the injection of high-energy carriers into the gate oxide, which results in interface state generation and oxide trap formation. This phenomenon is particularly pronounced when a high gate-to-drain voltage (*V*_gd_) accelerates carriers within the channel, leading to impact ionization near the drain region [254]. Under conditions where *V*_g_ ≈ *V*_d_, energetic carriers acquire sufficiency energy to surmount the potential barrier and become trapped within the oxide, leading to progressive degradation of the channel and gate dielectric. Conversely, in scenarios where *V*_g_ < *V*_d_, impact ionization results in additional charge carriers (holes in an n-channel MOSFET), further exacerbating *I*_*g*_ and accelerating oxide wear-out.

The long-term impact of hot-carrier degradation manifests as a gradual shift in key electrical parameters, including *V*th, S.S., and *µ* [254]. These changes degrade device performance and reliability, ultimately leading to failure in high-speed and high-power applications. To mitigate these effects, advanced gate stack engineering approaches, such as optimizing the doping profile of the channel and incorporating strain engineering techniques, have been explored [253]. These strategies effectively reduce the probability of high-energy carrier generation and enhance device robustness against hot-carrier degradation.

### Time-Dependent Dielectric Breakdown

Time-dependent dielectric breakdown (TDDB) is a critical reliability concern in MOSFETs, arising from the progressive degradation of the gate oxide due to prolonged exposure to high electric fields. As stress-induced defects accumulate within the dielectric, the formation of conductive percolation paths becomes increasingly probable, ultimately leading to catastrophic breakdown [255]. The rate at which TDDB occurs is strongly dependent on the magnitude of the applied electric field, with empirical studies suggesting a field-dependent lifetime model that follows an exponential decay relationship. Experimental data and empirical models suggest that dielectric lifetime (*τ*) scales inversely with field strength following an *E*-model or a *√E*-model dependence [260].

High-*K* dielectric materials, such as hafnium oxide (HfO_2_), zirconium oxide (ZrO_2_), and aluminum oxide (Al_2_O_3_), have been widely adopted to maintain high gate capacitance while increasing the physical thickness of the dielectric, thereby reducing direct tunneling leakage. However, TDDB remains a significant challenge in aggressively scaled devices, necessitating further advancements to extend the operational lifespan of ultra-scaled transistors. One approach to mitigating TDDB degradation is oxygen vacancy suppression, as high-*K* materials inherently contain a high density of oxygen vacancies, which act as electron trap centers and accelerate breakdown [255]. To address this, nitridation (N-incorporation) has been introduced, where nitrogen is incorporated into HfO_2_ to suppress oxygen vacancies and reduce defect density, leading to the development of nitrided high-*K* materials like HfSiON [261, 262]. Another strategy is interface engineering, as the interface between high-*K* dielectrics and the Si substrate plays a crucial role in TDDB reliability. A rough or defect-rich interface leads to increased trap states and accelerates breakdown, necessitating interfacial layer (IL) optimization. By incorporating an ultra-thin SiO_2_ layer between the high-*K* dielectric and Si substrate, defect density can be reduced, improving TDDB reliability while maintaining gate control [263]. However, careful optimization is required, as excessive IL thickness may degrade gate capacitance. In addition, plasma treatment using O_2_ or N_2_ can effectively passivate interface defects by reducing dangling bonds at the Si/high-*K* interface, further enhancing dielectric reliability and extending transistor lifespan.

### Negative Bias Temperature Instability

Negative bias temperature instability (NBTI) primarily affects p-channel MOSFETs (pMOSFET) under prolonged negative gate bias conditions. The degradation mechanism involves the generation of interface states at the Si/SiO_2_ interface and the trapping of holes within preexisting oxide defects. Over time, these phenomena result in a shift in the *V*th, leading to performance degradation and increased power consumption in CMOS circuits. The severity of NBTI-induced degradation is highly dependent on stress duration and operating temperature, following a power-law relationship with stress duration. Recovery effects, observed upon stress removal, indicate a partial reversal of the degradation, suggesting a dynamic equilibrium between trap generation and passivation. Advanced gate stack engineering, including the incorporation of nitrogen-passivated high-*K* dielectrics and optimization of hydrogen termination at the Si/SiO_2_ interface, has been proposed as an effective means of mitigating NBTI effects. These approaches enhance the robustness of the gate dielectric and reduce the rate of charge trapping, thereby improving the long-term reliability of pMOSFET devices.

### Time-Dependent Device-to-Device Variation

As MOSFET dimensions continue to shrink, stochastic variations in the fabrication process introduce significant variability in device performance. Time-dependent device-to-device variation (TDDV) arises from the random nature of oxide trap generation and removal, leading to unpredictable shifts in electrical characteristics across a device population. This variability is particularly pronounced in advanced semiconductor nodes, where statistical fluctuations in the number and spatial distribution of oxide traps introduce challenges in ensuring uniform circuit behavior.

To address this issue, Wani et al*.* [264] used alkali metal fluirudes as dielectric capping layers, including lithium fluoride (LiF), sodium fluoride (NaF), and potassium fluoride (KF) dielectric capping layers, to mitigate the environmental impact of oxygen and water exposure. Among them, the LiF dielectric capping layer significantly improved the transistor performance, specifically in terms of enhanced field-effect mobility from 74 to 137 cm^2^ V⁻^1^ s⁻^1^, increased current density from 17 to 32.13 μA μm⁻^1^ at a drain voltage of *V*_d_ of 1 V. Additionally, experimental studies have demonstrated that oxide trap dynamics contribute to random telegraph noise (RTN)-like fluctuations, resulting in increased *V*th variations and degraded drive current uniformity. As illustrated in Fig. 12, measurements of device performance under negative gate bias conditions reveal a broad statistical dispersion, indicating the impact of trap-assisted processes on overall device stability. Addressing TDDV requires improvements in process uniformity, enhanced defect management strategies, and the development of predictive models that account for stochastic variations in oxide trap formation.

### Future Perspectives on Reliability Enhancement

To ensure the continued scalability and performance of MOSFETs, a comprehensive approach to reliability enhancement must be pursued. Several key strategies have been identified as essential for mitigating degradation mechanisms and extending device lifetime. First, advancements in material engineering and process optimization are critical to improving the reliability of gate dielectrics. The development of novel high-*K* materials with enhanced dielectric robustness, coupled with advanced defect passivation techniques, can significantly reduce the impact of TDDB and NBTI. Additionally, optimizing channel doping profiles and strain engineering techniques can mitigate the effects of hot-carrier degradation, thereby enhancing device performance under high-field operation. Second, the integration of predictive reliability models is essential for accurately assessing degradation trends and optimizing device design. Physics-based models that capture the complex interactions between charge trapping, interface state generation, and dielectric breakdown can provide valuable insights into failure mechanisms, enabling the development of more resilient transistor architectures. Furthermore, machine learning-assisted predictive techniques have the potential to enhance reliability forecasting, facilitating early failure detection and proactive reliability management. Lastly, circuit-level design innovations must be explored to compensate for device-to-device variations and ensure robust system operation. Adaptive biasing schemes, error correction techniques, and dynamic compensation circuits can mitigate the impact of stochastic variability, enabling the implementation of reliable and energy-efficient semiconductor technologies.

To address how classical reliability concerns manifest in emerging materials, it is important to examine how these degradation and variability mechanisms influence novel semiconductors such as 2D materials, perovskites, and Mott insulators. Due to their atomic-scale thickness and high surface-to-volume ratio, 2D materials are highly susceptible to environmental interactions, charge trapping at the dielectric interface, and contact instability [265]. For example, threshold voltage drift and hysteresis under prolonged gate bias have been widely reported and are primarily attributed to interface trap generation and ambient-induced doping effects. Additionally, contact degradation due to Fermi level pinning and metal diffusion remains a key challenge. Several studies have proposed strategies to mitigate these instabilities. For instance, Ghosh et al. demonstrated through simulation that interface and border traps in monolayer MoS_2_ FETs induce charge trapping effects, leading to hysteresis and threshold voltage shifts that are strongly dependent on the gate sweep rate [266]. To address these effects, they emphasized the need for careful interface engineering and dielectric selection. Knobloch et al. systematically investigated the electrical stability of 2D material-based FETs and revealed that threshold voltage drift and hysteresis are primarily driven by border trap dynamics at the dielectric interface. Through experimental studies on graphene FETs with amorphous gate dielectrics, they demonstrated that careful tuning of the Fermi level can effectively suppress trap-related instabilities. Their findings underscore the importance of optimizing the gate dielectric environment and engineering a high-quality semiconductor–insulator interface to ensure long-term operational reliability in 2D transistors [267].

Analogous reliability concerns including TDDB- and NBTI-like instabilities have also emerged in perovskite-based electronic and optoelectronic devices through distinct physical origins. In particular, metal halide perovskites exhibit field- and light-induced ion migration (e.g., iodide, methylammonium), which leads to dynamic trap formation, phase segregation, and hysteresis—phenomena that mimic or exacerbate TDDB- and NBTI-like instabilities [268, 269]. Furthermore, the soft ionic lattice of perovskites makes them more susceptible to electric field-induced degradation and time-dependent variability under operational stress [270]. To mitigate these effects, recent studies have proposed material- and interface-level solutions such as: 1) *i*on trap passivation via incorporation of fullerene derivatives or small molecules, 2) compositional engineering using mixed-cation or mixed-halide strategies to suppress mobile ions, and 3) interfacial barrier layers (e.g., Al_2_O_3_, NiO_x_) that block ion migration and improve dielectric integrity [269, 271]. These efforts highlight that while the failure mechanisms differ in origin from those in traditional semiconductors, the need for robust reliability modeling and targeted mitigation remains equally critical in perovskite systems.

Mott materials, known for their strongly correlated electron systems, exhibit unique degradation behaviors distinct from conventional semiconductors. One major reliability issue is field-induced dielectric breakdown, where an abrupt Mott gap collapse leads to a sudden insulator-to-metal transition (IMT), causing unpredictable conductivity changes [272]. Additionally, in resistive switching devices, filament formation and overgrowth during repeated cycling result in threshold voltage variability and potential short circuits [182]. Unlike traditional failure modes like TDDB or HCD, Mott systems present challenges such as nonlinear switching dynamics, device-to-device variability, and irreversible damage from localized transitions. To address these, researchers have explored: Interface engineering (e.g., buffer layers, gating dielectrics) to moderate electric fields and suppress abrupt transitions [272, 273], device design tuning (e.g., series resistors, geometry control) to stabilize switching behavior [182], and pulse profile optimization and defect control to limit filament growth and improve repeatability [182]. These strategies are essential to enable reliable and scalable Mott-based memory and logic devices.

In conclusion, addressing reliability challenges in MOSFET devices requires a multifaceted approach that integrates material advancements, process optimizations, and predictive modeling strategies. As transistor technology progresses toward sub-3 nm nodes and beyond, ensuring device reliability will remain a fundamental challenge, necessitating continuous innovation in semiconductor reliability engineering.

## Perspectives and Outlook

### Key Perspectives on Addressing Device Limitations

**Fig. 13:**
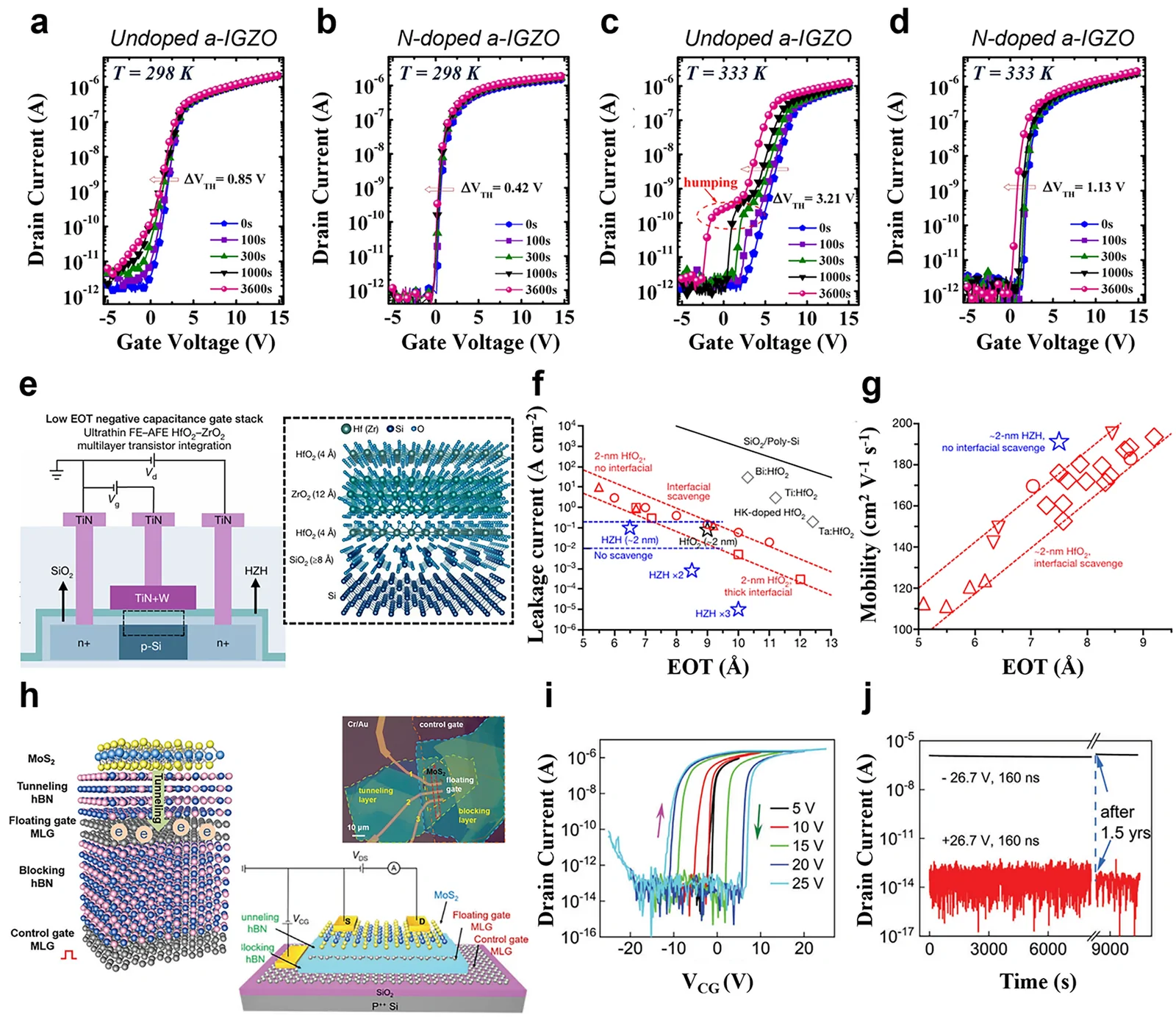
Strategies for BTI mitigation, leakage suppression, and high-performance switching. **a**–**d** Transfer curves of a-IGZO transistors under various bias stress conditions, demonstrating Δ*V*th reduction through N doping. Reproduced with permission from [276]. Copyright 2013, AIP Publishing. **e**–**g** Device performance benefits of ultra-thin mixed-ferroic HfO_2_–ZrO_2_ (HZH) gate stacks for leakage current suppression. **e** Schematic of a transistor device integrating a 2-nm HZH gate stack (HZH fluorite–structure multilayer). **f** Leakage current versus EOT for HZH multilayer gate stacks (blue), benchmarked against reported high-*K* metal gate (HKMG) literature [280], including interlayer-scavenged 2-nm HfO_2_ (red), high-*K* doped HfO_2_ (gray), and SiO_2_/poly-Si (black). HZH achieves the lowest reported leakage for a 6.5-Å EOT MOS capacitor [280], comparable to higher-EOT 2-nm HfO_2_ due to the preservation of the interfacial SiO_2_ layer. Reproduced with permission from [277]. Copyright 2022, Springer Nature. **g** Carrier mobility versus EOT for long-channel transistors integrating 2-nm HZH (blue) compared to industry-reported transistors with 2-nm HfO_2_ (red) [281]. HZH exhibits higher mobility by achieving ultra-thin EOT without requiring interfacial scavenging. Implementation of a floating gate transistor system for enhanced switching stability and high-performance characteristics: **h** Schematic illustration of the floating gate architecture using MoS_2_ as the channel material, tunneling hBN as the insulator, and a control gate for precise charge modulation. **i** Transfer characteristics across varying control gate biases. **j** Long-term stability measurements demonstrating excellent charge retention after programming/erasing cycles, indicating the potential for reliable, high-performance operation in advanced electronic devices. Reproduced with permission [279]. Copyright 2024, Wiley

The continuous downscaling of semiconductor devices has introduced significant challenges related to bias thermal instability (BTI), leakage currents, and switching performance beyond conventional silicon-based MOSFETs. To overcome these limitations, novel materials, device architectures, and optimization strategies must be explored. This section discusses key approaches for mitigating BTI, tackling leakage issues, and realizing high-performance switching characteristics, highlighting recent advancements in oxide semiconductor transistors, hybrid dielectric engineering, and floating gate transistor architectures. Figure 13 provides an overview of various strategies addressing these challenges.

#### Mitigating Bias Thermal Instability

BTI presents a significant reliability challenge in TFTs, where prolonged bias stress leads to a shift in *V*th, impacting long-term device performance. This degradation is commonly observed in amorphous oxide semiconductors such as a-IGZO, where charge trapping at defect sites contributes to *V*th instability. Figure 13a–d shows the transfer characteristics of undoped and nitrogen (N)-doped a-IGZO transistors under different bias stress conditions at room temperature (298 K) and elevated temperature (333 K) [276]. The data reveal a significant improvement in *V*th stability in the N-doped samples, as indicated by the reduced Δ*V*th. Specifically, at higher temperatures, undoped a-IGZO experiences pronounced *V*th shifts and "humping" effects due to increased charge trapping. In contrast, *N* doping effectively suppresses these phenomena by modulating defect states and mitigating carrier trapping at the semiconductor–dielectric interface. To further enhance BTI resilience, passivation techniques and interface engineering strategies must be employed. The introduction of high-mobility oxide semiconductors, coupled with optimized doping strategies, presents a promising pathway for stable and high-performance thin-film transistor technologies.

#### Tackling Leakage Issues

One of the persistent challenges in aggressively scaled transistors is leakage current through the gate dielectric. As transistors shrink, the need for an ultra-thin gate oxide layer increases, leading to higher electric fields and enhanced quantum mechanical tunneling, which significantly raises *I*_*g*_. Traditional high-*K* materials such as Al_2_O_3_ offer improved capacitance but often suffer from higher leakage currents due to interface roughness and charge scattering at defect sites. To address these limitations, hybrid dielectric stacks incorporating polymer-based insulators, such as PMMA (polymethyl methacrylate), were used as a promising alternative for leakage suppression [157]. When combined with Al_2_O_3_, the hybrid dielectric system offers a more controlled interfacial electric field, which helps to suppress defect-assisted conduction and improve the overall stability of the transistor. Experimental findings have demonstrated the advantages of this approach in two key aspects. First, the integration of a PMMA layer results in a significant improvement in field-effect mobility, indicating enhanced carrier transport properties. This is attributed to the suppression of charge trapping at the dielectric interface, which is a common issue in conventional Al_2_O_3_-based gate stacks. Second, subthreshold slope measurements across different applied biases confirm that the PMMA/Al_2_O_3_ dielectric system effectively reduces *I*_g_. The hybrid structure provides better electrostatic control, leading to improved transistor operation and greater energy efficiency.

Recent study by Cheema et al*.* [277] introduces a promising alternative by leveraging HfO_2_–ZrO_2_ (HZH) superlattice heterostructures as a gate dielectric (Fig. 13e). Unlike conventional HfO_2_ stacks, which rely on aggressive interfacial engineering to reduce equivalent oxide thickness (EOT), HZH achieves ultra-thin dielectric scaling (approximately 20 Å in physical thickness, with an EOT as low as 6.5 Å) without requiring SiO_2_ scavenging. This structural advantage helps maintain a clean interface, thereby minimizing defect-related conduction and preserving transistor performance. One of the key benefits of HZH superlattices is their ability to significantly suppress *I*_g_. As shown in Fig. 13f, conventional HfO_2_-based dielectrics suffer from increased leakage due to interfacial SiO_2_ scavenging, which introduces defects and roughness. In contrast, HZH achieves comparable EOT without scavenging, leading to substantially lower leakage current, improved reliability, and better power efficiency. In addition to leakage suppression, HZH structures also maintain high carrier mobility, which is crucial for high-speed transistor operation (Fig. 13g). While conventional HfO_2_ gate stacks exhibit mobility degradation at lower EOT due to charge scattering and defect trapping, HZH preserves a smooth, defect-free interface, thereby enhancing carrier transport efficiency.

These findings highlight the potential of multi-dielectric stacks in reducing leakage current while maintaining high performance. By combining different dielectric materials, these engineered stacks provide a scalable and energy-efficient solution for next-generation semiconductor devices. As device scaling progresses, continued advancements in defect engineering and interfacial passivation will be essential in further optimizing these hybrid dielectric structures.

#### Realizing High-Performance Switching beyond Si MOSFETs

To transcend the intrinsic limitations of Si MOSFETs and realize next-generation high-performance switching, future research must focus on the strategic integration of emerging semiconductors—including 2D materials, halide perovskites, oxide semiconductors, etc.—with well-established but continuously evolving dielectric materials. While high-*K* dielectrics, ferroelectrics, and vdW dielectrics have been extensively studied, their role in enabling ultra-scaled, low-power electronics must be re-evaluated in the context of novel semiconductor systems. In particular, the interplay between 2D semiconductors and dielectrics can lead to unique electronic and interfacial phenomena that go beyond conventional silicon-based systems.

A compelling demonstration of this principle is the floating gate transistor architectures for achieving high-performance and reliable switching characteristics [222, 278, 279]. Figure 13h–j illustrates the implementation of a floating gate transistor system utilizing MoS_2_ as the channel material, with a multilayer hBN tunnel barrier and a control gate for precise charge modulation [279]. The schematic representation in Fig. 13h highlights the role of tunneling and blocking hBN layers in charge trapping and retention. This configuration enables effective charge storage and modulation, making it highly suitable for nonvolatile memory and logic applications. The transfer characteristics presented in Fig. 13i demonstrate robust switching behavior across varying control gate voltages, with distinct hysteresis effects indicative of strong charge storage capabilities. Additionally, long-term stability measurements in Fig. 13j confirm excellent charge retention even after extensive programming/erasing cycles, reinforcing the reliability of this architecture for high-endurance applications.

Moving forward, the realization of commercially viable next-generation electronics will hinge on the ability to engineer semiconductor–dielectric interfaces with atomic precision, optimize dielectric–semiconductor band alignments for enhanced charge modulation, and develop scalable fabrication techniques for integrating these materials into large-area, high-density device architectures. By strategically leveraging the synergy between advanced semiconductors and carefully designed dielectric environments, the limitations of conventional Si-based electronics can be systematically overcome, paving the way for energy-efficient, high-speed, and ultra-scaled electronic systems.

### Outlook for Transistor Manufacturing

**Fig. 14:**
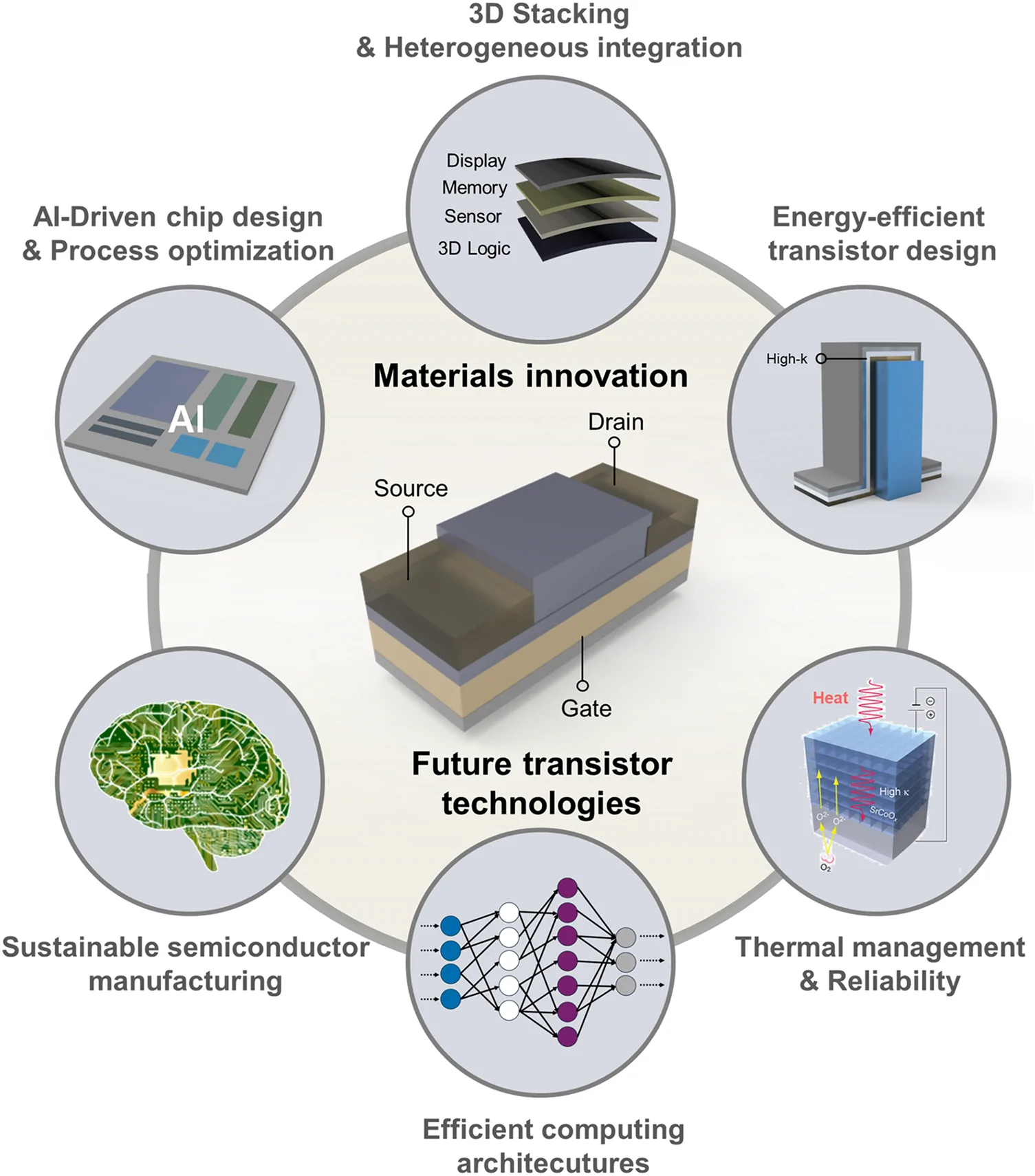
Perspectives and outlook for future transistor technologies with materials innovation. Addressing key challenges in the semiconductor industry, including bias thermal instability, leakage issues, and high-performance switching. Advancing thermal management solutions and 3D stacking architectures to enhance device performance and integration density. Exploring emerging computing paradigms and integration with AI to meet evolving technological demands. Sustainable manufacturing and energy-efficient processing to ensure long-term industry viability. Reproduced with permission from [282]. CC.BY.4.0

The continued miniaturization of transistors has driven semiconductor technology toward fundamental scaling limits, necessitating new approaches beyond conventional planar CMOS scaling. As power consumption, interconnect resistance, and thermal constraints become increasingly dominant challenges, alternative solutions such as 3D stacking, energy-efficient transistor architectures, and AI-driven computing paradigms are emerging as the foundation for next-generation semiconductor devices. The 3D stacking architecture enables the industry to move beyond transistor density scaling by integrating heterogeneous computing units, reducing interconnect delays, and improving system-level efficiency. In parallel, energy-efficient transistor designs, including steep-slope transistors and ultra-low-*V*th devices, aim to overcome the thermionic S.S. limit, reducing operating voltages and minimizing power dissipation in high-performance applications. Thermal management remains a critical challenge as chip densities increase and AI accelerators demand sustained computational loads. Novel passive and active cooling techniques, high-thermal-conductivity materials, and AI-driven dynamic thermal regulation are being explored to ensure long-term reliability and performance stability in 3D stacked and high-power semiconductor devices. The integration of neuromorphic computing, memory-in-logic architectures, and AI-optimized semiconductor platforms is transforming the role of hardware in intelligent computing beyond power and thermal considerations. AI-driven semiconductor architectures are also introduced how data are processed, accelerating inference efficiency while reducing energy overhead. The convergence of AI, 3D integration, and ultra-low-power transistor architectures marks a fundamental shift in semiconductor innovation. By addressing interconnect resistance, process compatibility, and computational efficiency, the semiconductor industry is set to transition toward highly adaptive, scalable, and intelligent computing architectures that extend beyond conventional logic scaling (Fig. 14).

#### Three-Dimensional (3D) Stacking Architectures: Heterogeneous Integration

The increasing demand for higher computational performance, energy efficiency, and reduced form factors has driven the transition from planar CMOS scaling toward 3D stacking architectures. While planar transistor scaling has historically enabled continuous improvements in density, interconnect delays, power dissipation, and heat management constraints have become major limiting factors. To overcome these challenges, 3D integration has emerged as a transformative approach, enabling enhanced interconnect efficiency, functional diversification, and heterogeneous system integration. The semiconductor industry has actively invested in 3D IC packaging and stacking methodologies to maintain performance scaling beyond conventional transistor density limits [283, 284]. Advanced technologies, including Intel’s Foveros, TSMC’s 3D Fabric, and system-on-integrated-chip (SoIC) architectures utilize chiplet stacking, hybrid bonding, and high-density interconnect solutions to enhance efficiency [284–287]. These approaches facilitate higher bandwidth, reduced latency, and improved power–performance trade-offs, positioning 3D stacking as a crucial enabler of next-generation computing architectures. In addition, GAA-FETs are expected to dominate the sub-2 nm technology node, whereas by the 1.5 nm node-targeted around 2028-transistor architecture incorporating vertically stacked nMOS and pMOS channels are anticipated to emerge. These architectures aim to minimize footprint via vertical integration and employ shared gate configurations to enable tighter control. Such innovations are projected to overcome fundamental scaling bottlenecks, paving the way for novel three-dimensional device concepts that may define the next paradigm in semiconductor technology. Despite these advancements, material and process challenges remain, particularly in interconnect resistance, thermal dissipation, and CMOS compatibility. Future research must focus on novel interconnect materials, optimized stacking methodologies, and monolithic 3D integration to fully exploit the potential of 3D semiconductor architectures.

The evolution of 3D integration has progressed from 2D ICs to 2.5D and ultimately 3D ICs, introducing higher transistor densities and improved interconnect performance. TSV technology has been the most widely adopted approach for enabling vertical interconnects between stacked layers. However, TSV pitch scaling below 10 μm remains challenging due to fabrication complexity and parasitic capacitance [288]. Hybrid bonding has emerged as an alternative, offering finer interconnect pitches, reduced resistive losses, and improved power efficiency, though further optimization is required to improve interface reliability and manufacturing yield [289]. Monolithic 3D integration, which involves sequential device fabrication on a single wafer, eliminates TSV-related constraints and allows ultra-dense stacking, but requires low-temperature processing strategies to preserve device integrity. As parallelized 3D architectures gain traction, the International Roadmap for Devices and Systems (IRDS) anticipates a shift toward monolithic 3D integration, where individual transistor layers are vertically stacked within the same wafer [290]. This approach enables high interconnect density, reduced parasitic capacitance, and improved energy efficiency, overcoming the limitations of traditional 3D IC packaging [291–293].

While 3D stacking architecture offers a scalable alternative to planar transistor scaling, their widespread adoption depends on resolving key material challenges, particularly in interconnect resistivity, thermal management, and process integration. The limitations of Cu-based interconnects, including electron scattering, electromigration, and parasitic resistance from diffusion barriers, are becoming increasingly critical as transistor density scales beyond sub-10 nm nodes. Alternative conductors such as 1D metallic nanowires, 2D layered metals, Weyl semimetals, and ultra-thin Ru/Co interconnects are under investigation to address these constraints. These materials offer lower resistivity at the nanoscale, enhanced carrier mobility, and improved electromigration resistance, making them viable candidates for next-generation BEOL integration. The transition to vertically stacked architecture inherently introduces self-heating effects and increased thermal resistance due to the reduced surface area available for heat dissipation. Advanced thermal interface materials (TIMs), dielectric heat spreaders, and engineered heat dissipation pathways are being explored to improve thermal reliability in HPC environments. Additionally, as monolithic 3D stacking gains interest, maintaining process compatibility between sequentially fabricated layers remains a fundamental challenge. Innovations in low-temperature processing, atomic-scale interface engineering, and defect-free material deposition are required to enable high-performance, vertically integrated logic-memory systems.

The benefits of 3D stacking extend beyond interconnect scaling, offering opportunities for heterogeneous integration with emerging transistor architectures that surpass the limitations of FinFET and GAAFET technologies. Two-dimensional material-based FETs, such as those using MoS_2_ or WSe_2_, allow ultra-thin channel structures that mitigate short-channel effects in vertically stacked logic layers. FeFETs provide nonvolatile memory functionalities within logic devices, reducing static power consumption and enabling in-memory computing paradigms [294]. Negative capacitance FETs (NC-FETs) leverage ferroelectric layers to improve switching efficiency and achieve sub-60 mV/decade operation, enhancing overall energy efficiency. As monolithic 3D integration continues to advance, the ability to incorporate these novel transistor technologies will further extend the functional and performance benefits of 3D stacked semiconductor architectures.

Despite its transformative potential, 3D stacking architecture must overcome key challenges in CMOS process compatibility, thermal dissipation, and heterogeneous integration. The deposition, patterning, and etching of emerging conductors and dielectrics must be refined to ensure seamless compatibility with existing BEOL workflows. Self-heating effects in vertically stacked structures necessitate the development of advanced thermal management solutions, while interlayer thermal coupling must be precisely engineered to maintain performance consistency in stacked logic-memory systems. The integration of 3D monolithic memory-in-logic architectures could accelerate AI inference tasks while significantly reducing power consumption. Furthermore, non-von Neumann computing paradigms remain an area of active research within 3D architecture, particularly for neuromorphic and edge AI applications. By addressing these challenges, 3D stacking architectures will play a pivotal role in defining the next era of semiconductor innovation, particularly in the heterogeneous integration of AI-driven computing, high-performance logic-memory convergence, and ultra-dense chiplet architectures.

#### Pushing Energy Efficiency: Super Low-Vth & Sub-60 mV dec⁻1 S.S.

As HPC continues to expand, improving the energy efficiency of semiconductor devices has become a critical challenge. However, conventional CMOS technology is fundamentally constrained by the S.S. limit of 60 mV dec⁻^1^ at room temperature, restricting further reductions in operating voltage and impeding ultra-low-power design. Without overcoming this limitation, power consumption in HPC systems, such as AI accelerators and data centers, will continue to rise, exacerbating energy efficiency concerns. To address this challenge, researchers are exploring new transistor architectures featuring super low-*V*th transistors and sub-60 mV dec⁻^1^ S.S. devices. Super low-*V*th transistors operate at lower voltages, significantly reducing power consumption [295]. However, lowering the *V*th also increases leakage current, leading to higher static power dissipation and potential thermal issues. Moreover, process-induced variations in *V*th introduce unpredictability in transistor performance, particularly in memory applications such as SRAM, where stability is critical. To mitigate these challenges, various techniques, including high-*K* gate dielectrics, multi-*V*th design strategies, and adaptive biasing techniques, are being developed to balance low operating voltage and leakage control [296]. Beyond lowering the *V*th, a more fundamental approach involves overcoming the S.S. limit of conventional MOSFETs to enable ultra-low-power switching. This has led to intense research into alternative transistor designs such as tunnel field-effect transistors (TFETs), NC-FETs, and Dirac-source field-effect transistors (DSFETs). TFETs utilize band-to-band tunneling to enable sub-thermal S.S., while NC-FETs leverage negative capacitance effects from ferroelectric materials to amplify the internal gate voltage, reducing S.S. beyond the 60 mV dec⁻^1^ limit. However, both technologies present practical challenges: TFETs suffer from low on-current, and NC-FETs require precise capacitance matching for stable operation. Recently, DSFETs have emerged as a promising alternative, utilizing Dirac electrons’ linear dispersion properties to address the limitations of TFETs, offering high *I*_on_ and efficient low-voltage operation. A key enabler for these next-generation transistors is the integration of 2D materials. 2D materials, such as TMDs, exhibit atomic-scale thinness, high carrier mobility, and defect-free van der Waals surfaces, making them highly suitable for steep-slope transistors [297]. Additionally, van der Waals heterojunctions allow for ultra-clean interfaces, enhancing tunneling efficiency in TFETs and stabilizing negative capacitance effects in NC-FETs. Recent studies suggest that 2D material-based DSFETs could significantly lower power consumption, making them a strong candidate for future ultra-low-power electronics [298].

These innovations have broad application potential across multiple domains. AI accelerators and neuromorphic computing require energy-efficient semiconductor devices to enhance computational speed while minimizing power dissipation. Likewise, low-power IoT and wearable devices demand efficient transistors to extend battery life and enable sustainable operation. Edge computing and battery-powered systems can benefit from ultra-low-power transistors, improving performance while reducing energy consumption. The realization of sub-60 mV dec⁻^1^ transistor technologies necessitates seamless integration with existing CMOS fabrication processes while simultaneously enhancing device performance. Advances in TFETs, NC-FETs, and DSFETs continue to drive progress in energy-efficient semiconductor design, with ongoing research into novel device architectures and material systems expanding the frontiers of ultra-low-power electronics. These developments are expected to play a critical role in shaping the next generation of high-performance, energy-efficient computing technologies.

#### Enhancing Thermal Tolerance in Integrated Chips

As AI accelerators, HPC and data centers continue to scale, the demand for greater computational power has led to a substantial increase in power consumption. This, in turn, has introduced significant thermal management challenges, particularly in 3D stacked chip architectures, where the vertical integration of multiple layers restricts heat dissipation. Unlike traditional planar chips, which can efficiently dissipate heat through the surface, 3D stacked chips suffer from heat accumulation within the structure, leading to the formation of thermal hot spots. As the number of stacked layers increases, heat becomes trapped, degrading performance, increasing power leakage, and reducing overall device reliability. This issue is particularly severe in high-power computing applications such as AI accelerators and neuromorphic chips, where excessive heat can cause thermal throttling, limiting computational efficiency [299]. Given these challenges, effective thermal management strategies are critical to ensuring the stability and longevity of 3D integrated circuits (3D ICs).

To mitigate heat accumulation in 3D stacked chips, researchers have explored both passive material-based solutions and active cooling techniques. Passive cooling approaches focus on integrating high-thermal-conductivity materials to enhance heat dissipation within the stack. Graphene and CNTs [300] have emerged as promising heat-spreading materials, with thermal conductivities exceeding 3000 W m⁻^1^ K⁻^1^, enabling efficient channeling of heat away from hot spots. Diamond-based thermal interface materials (TIMs) [301], known for their exceptional thermal conductivity, have been incorporated into TSVs to facilitate vertical heat dissipation. Additionally, silicon carbide (SiC) interposers have been explored as a superior alternative to conventional silicon-based interposers, offering improved thermal spreading between stacked dies. Studies indicate that these material innovations can reduce hot spot temperatures by up to 30%, significantly improving thermal performance in multilayer semiconductor structures. However, while these materials enhance passive heat dissipation, they may not be sufficient for high-power applications, where more active cooling methods are required.

To address this limitation, researchers have turned to active cooling techniques that can dynamically regulate heat dissipation in real time. One such approach is microfluidic cooling, which integrates coolant channels directly within the chip stack to enable localized liquid cooling at critical regions. Studies have shown that microfluidic cooling can reduce peak chip temperatures by more than 40% compared to conventional air-cooling methods, making it particularly suitable for data centers and HPC environments, where large-scale heat dissipation is required. Another promising technique is thermoelectric cooling (TEC), which leverages the Peltier effect to transfer heat from a hot region to a cooler one using an electric current. TEC is particularly effective for chip-scale applications, where precise, localized cooling is needed. Hybrid cooling solutions, which integrate both microfluidic cooling and TEC, have demonstrated significant improvements in heat dissipation efficiency, making them viable for next-generation AI processors and high-power semiconductor devices.

Beyond hardware-based solutions, thermal-aware design strategies play a crucial role in managing heat in 3D stacked chips. Dynamic thermal management (DTM) techniques adjust clock frequencies and operating voltages based on real-time temperature monitoring, preventing localized overheating. Additionally, thermal-aware task scheduling optimally distributes workloads across multiple chip layers, balancing heat generation to avoid excessive thermal buildup. Machine learning-based thermal prediction models further enhance these strategies by enabling real-time hot spot forecasting, allowing preemptive adjustments to cooling mechanisms. Support vector regression (SVR) models, for instance, have been demonstrated to predict hot spot temperatures with an accuracy of 0.6 K, facilitating more precise and adaptive thermal regulation in 3D ICs with up to 28 layers. These AI-driven approaches complement passive and active cooling solutions by ensuring that thermal management is not just reactive but also proactively optimized in response to workload demands [299]. The implementation of these advanced thermal management strategies is expected to transform key application areas. AI accelerators and neuromorphic computing chips, which require sustained high-performance operation, will benefit from enhanced thermal regulation, reducing the risk of thermal-induced performance degradation. Data centers and HPC environments, where power efficiency and large-scale heat dissipation are primary concerns, can integrate these cooling technologies to lower infrastructure costs while improving computational efficiency. Moreover, AI-driven real-time thermal optimization offers a compelling pathway for autonomous thermal management, reducing energy overhead associated with cooling systems.

Looking ahead, the continued advancement of 3D semiconductor packaging will depend heavily on overcoming thermal bottlenecks through multifaceted solutions. The integration of high-thermal-conductivity materials, active cooling methods, and AI-driven thermal management techniques presents a viable strategy for sustaining Moore’s law while ensuring efficient energy management in next-generation computing architectures. Future research into low-temperature bonding techniques, advanced interconnect designs, and embedded cooling solutions will be instrumental in fully unlocking the potential of 3D stacked chips, paving the way for more thermally efficient and scalable semiconductor technologies.

#### Meeting the Demand for New Functionalities

As AI systems become increasingly complex, the demand for more efficient computing architecture extends beyond conventional transistor scaling. Traditional von Neumann computing architecture suffers from energy inefficiency, memory bandwidth limitations, and processing delays, which have become particularly problematic for real-time AI applications such as autonomous systems, robotics, and bio-inspired computing [302]. To address these challenges, neuromorphic computing has emerged as a promising alternative, leveraging brain-inspired architectures that integrate memory and processing to achieve highly efficient, low-power computing. However, intelligence in biological systems is not solely about computation; it also involves real-time sensory perception and adaptive learning. To bridge this gap, neuromorphic computing must be complemented by neuromorphic sensory devices, which mimic biological sensory systems to process data more efficiently at the hardware level.

Neuromorphic hardware is fundamentally different from traditional digital processors, as it relies on event-driven, massively parallel, and in-memory computing principles to reduce energy consumption and improve processing efficiency. Instead of sequential, clock-driven operations, neuromorphic systems process information dynamically, responding to stimuli in a manner emulating biological synapses and neurons. This functionality is largely enabled by emerging materials such as halide perovskites, ferroelectric oxides, and Mott insulators, which exhibit nonvolatile memory states, tunable resistance switching, and adaptive electrical responses—all of which are critical for replicating synaptic plasticity [303]. These material properties have facilitated the development of memristors, ferroelectric synapses, and ionic-based resistive switching devices, which provide multi-level resistance states essential for hardware-based learning and inference.

In parallel, neuromorphic sensory devices are revolutionizing the way machines perceive and interact with their environment [304]. Traditional CMOS-based sensors capture and process data continuously, leading to enormous data redundancy and excessive power consumption. In contrast, neuromorphic sensors operate on event-driven principles, mimicking biological sensory systems such as vision, touch, and olfaction. For instance, neuromorphic vision sensors, inspired by the human retina, detect changes in light intensity rather than capturing full-frame images at fixed time intervals, significantly reducing data transfer loads [305]. Similarly, neuromorphic tactile sensors emulate human skin by responding to pressure and texture changes with real-time electrical impulses, enabling energy-efficient haptic feedback in robotics. Bio-inspired chemical sensors, such as those mimicking the olfactory system, detect and classify complex chemical compositions with high specificity, offering potential applications in environmental monitoring and medical diagnostics. The convergence of neuromorphic computing and neuromorphic sensory devices creates a powerful synergy, allowing real-time perception, adaptation, and decision-making at the hardware level. Unlike conventional AI architectures, which rely on high-power computing clusters for inference and training, neuromorphic systems can perform these functions locally, within edge devices, using minimal energy. This is particularly relevant for applications such as autonomous robotics, brain–machine interfaces, and AI-driven prosthetics, where real-time response and low power consumption are crucial.

To fully leverage the potential of neuromorphic systems, several key challenges must be addressed. First, the scalability and manufacturability of neuromorphic materials must be improved to enable large-scale integration into semiconductor fabrication processes. While materials like halide perovskites and Mott insulators exhibit remarkable neuromorphic properties, their stability and compatibility with CMOS processing require further optimization. Second, neuromorphic computing architecture must be co-designed with AI algorithms to ensure that the hardware is fully utilized for practical applications. Unlike traditional deep learning models, which are optimized for digital hardware, neuromorphic AI requires dedicated software frameworks and learning paradigms that align with event-driven processing.

Looking ahead, neuromorphic computing and sensory devices represent a fundamental shift in how AI is realized at the hardware level. Future semiconductor architecture will increasingly move away from conventional digital logic toward bio-inspired, adaptive, and energy-efficient systems. The integration of neuromorphic transistors, 3D stacked memory, and event-driven sensory devices will enable AI systems that are not only computationally powerful but also capable of real-time perception and learning. As AI applications continue to expand into autonomous systems, bio-integrated electronics, and real-time edge computing, neuromorphic hardware will play a pivotal role in shaping the next generation of intelligent machines. Future research must focus on material optimization, system integration, and neuromorphic-native AI frameworks, ensuring that these emerging technologies can be seamlessly deployed in real-world applications.

#### Sustainable Manufacturing and Energy-Efficient Processing

The semiconductor industry is at the forefront of technological advancements but is also a sector with high energy consumption and environmental impact. Sustainable manufacturing and energy-efficient processing are among the most pressing challenges, requiring strategies that minimize carbon emissions, conserve resources, and reduce environmental footprints.

Recent studies have proposed various strategies to enhance the sustainability of semiconductor manufacturing processes. One critical approach is the development of energy-efficient transistor technologies. Conventional CMOS-based transistors have reached their physical limits, making further reductions in power consumption increasingly difficult. As an alternative, TFETs have gained attention. TFETs leverage quantum tunneling to operate at one-tenth the power of conventional CMOS devices while maintaining high performance, making them a key component in low-power applications. Additionally, innovations such as high-*K*/metal gate and FinFET architectures are being explored to further reduce power consumption and enhance performance [306].

Beyond transistor design, the adoption of eco-friendly materials in semiconductor fabrication is another significant aspect of sustainable manufacturing. Alternatives to silicon-based transistors, such as gallium nitride (GaN) and gallium oxide (Ga_2_O_3_), offer higher breakdown voltages and lower thermal resistance, making them well suited for energy-efficient power electronics [306]. At the same time, manufacturing processes are being refined to reduce the use of hazardous chemicals. For instance, EUV lithography is being utilized to decrease photoresist consumption and minimize waste production. The integration of smart manufacturing and automation is another essential strategy to reduce energy consumption in semiconductor processing. AI-driven optimization systems and IoT sensors enable real-time monitoring of manufacturing processes, preventing unnecessary resource waste. By implementing these smart factory technologies, major semiconductor companies like Samsung and Intel are optimizing their production lines to lower carbon emissions and enhance overall efficiency [306]. Furthermore, renewable energy adoption and waste recycling systems are emerging as fundamental components of sustainable semiconductor production. Companies are increasingly transitioning to solar and wind energy sources to power their fabs, thereby reducing their dependence on fossil fuels. Additionally, wastewater recycling systems are being implemented to mitigate the environmental impact of semiconductor fabrication. Notably, Intel has reported achieving over 90% water recycling rates in some of its facilities, demonstrating the feasibility of large-scale sustainable manufacturing initiatives.

To ensure long-term sustainability, the semiconductor industry must adopt a multifaceted approach that incorporates high-efficiency transistors, eco-friendly materials, process optimization, smart manufacturing technologies, and renewable energy utilization. Moving forward, semiconductor manufacturers must continue to innovate and invest in sustainable production techniques, balancing environmental responsibility with economic viability.

#### Integration with AI

The integration of AI with semiconductor technology represents a paradigm shift in computing, where hardware is no longer a passive executor of algorithms but an active enabler of intelligence. As AI workloads continue to grow in complexity, conventional computing architectures, particularly those based on the von Neumann model, are reaching their fundamental efficiency limits. The increasing energy consumption, memory bandwidth constraints, and latency associated with large-scale AI models necessitate a rethinking of how AI is embedded at the hardware level. Future semiconductor technologies must not only optimize traditional digital processing but also incorporate new computational paradigms that align with the principles of AI-driven computation, enabling higher efficiency, lower power consumption, and improved adaptability for emerging intelligent systems.

One of the most immediate and impactful areas of AI integration is processing-in-memory (PIM) and memory-centric computing, where data storage and computation occur within the same physical space to reduce energy-intensive data movement. Emerging memory technologies such as FeFETs, resistive RAM (RRAM), and phase-change memory (PCM) are leading this transition by enabling in-memory matrix operations that significantly accelerate AI inference. These materials, when integrated monolithically within semiconductor architectures, could enable near-zero latency computation, drastically reducing the power footprint of AI accelerators in both edge and cloud environments. Beyond performance improvements, the adoption of AI-centric memory architectures could redefine system-level computing paradigms, allowing hardware to be inherently optimized for deep learning workloads rather than retrofitted to accommodate them.

Alongside memory innovations, AI-optimized transistor architectures are emerging as a crucial driver of future efficiency gains. Ferroelectric materials and negative capacitance FETs (NC-FETs) offer the potential for sub-thermionic switching, reducing power consumption to levels previously unattainable with traditional CMOS. The integration of 2D semiconductors such as MoS₂ and WSe₂ into AI-specific transistor designs introduces new possibilities for dynamic threshold tuning, adaptive logic, and ultra-low-power processing, enabling energy-efficient AI computation even in resource-constrained environments [307, 308]. As AI applications become more pervasive in edge devices, from autonomous systems to wearables, these transistor-level optimizations will be essential in pushing AI beyond the confines of power-hungry data centers.

Beyond hardware optimizations, AI itself is becoming an integral tool for semiconductor design, process control, and device optimization. The application of AI-driven methodologies, such as neural architecture search for circuit optimization and machine learning-based process control, is transforming the way semiconductor devices are fabricated and tuned for performance. AI-enhanced design flows can accelerate the discovery of new transistor geometries, interconnect materials, and chip layouts, reducing the time and cost associated with traditional semiconductor research and manufacturing. As the semiconductor industry moves toward more heterogeneous and specialized architectures, AI-driven design automation will play a central role in optimizing chip performance, reliability, and scalability.

The long-term trajectory of AI-driven semiconductor innovation points toward increasing convergence between logic, memory, and intelligence at the hardware level [253, 274]. The rise of 3D stacking and monolithic integration will further enhance AI efficiency by minimizing communication bottlenecks between computing and memory layers, enabling architecture where AI processing is distributed across tightly integrated, high-bandwidth computing units. As AI models evolve toward self-learning and continuously adaptive systems, future semiconductor platforms will need to support real-time, low-power adaptation at both the material and circuit levels. The integration of AI and semiconductor technology is expected to revolutionize computational efficiency. The seamless integration of AI-optimized memory, adaptive transistors, and AI-driven chip design methodologies will create self-optimizing, intelligent computing systems that can dynamically adjust their operation based on workload demands. As AI applications expand beyond traditional domains into areas such as neuromorphic intelligence, bio-inspired computing, and autonomous reasoning, semiconductor architectures must evolve to become not just enablers of AI, but active participants in intelligent computation. The next decade will likely see AI-native semiconductor platforms, where materials, devices, and system architectures are co-designed to maximize AI performance, paving the way for ultra-efficient, autonomous, and scalable computing systems.
